# Supplementary figures and images for: Applying a new mold temperature control strategy to improve the tensile strength of thin wall products in injection molding processes
Source: PLoS One. 2025 Dec 5;20(12):e0337889. doi: 10.1371/journal.pone.0337889 (PMC12680320; doi:10.1371/journal.pone.0337889)

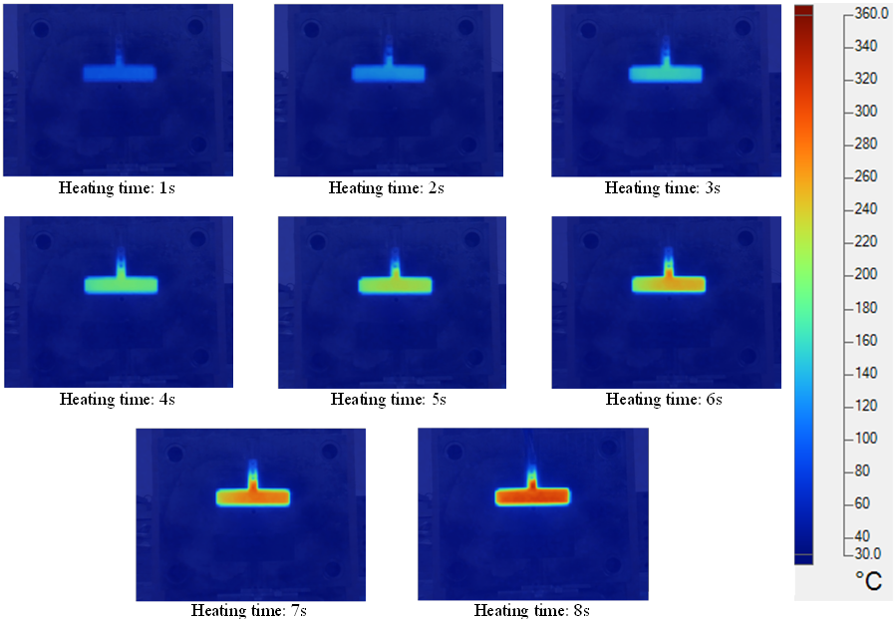

Supplement: S1 Appendix — Figure A1. Temperature distribution of Insert at the heating time from 1 s to 5 s with the gap (G) of 5 mm. This is the simulation results. Figure A2. Temperature distribution of Insert at the heating time from 1 s to 5 s with the gap (G) of 10 mm. This is the simulation results. Figure A3. Temperature distribution of Insert at the heating time from 1 s to 5 s with the gap (G) of 15 mm. This is the simulation results. Figure A4. Temperature distribution of a cavity plate with different heating times under a gap (G) of 5 mm. This is the experiment results. Figure A5. Temperature distribution of a cavity plate with different heating times under a gap (G) of 10 mm. This is the experiment results. Figure A6. Temperature distribution of a cavity plate with different heating times under a gap (G) of 15 mm. This is the experiment results. (ZIP) [file pone.0337889.s001.zip › Figure A6.png]

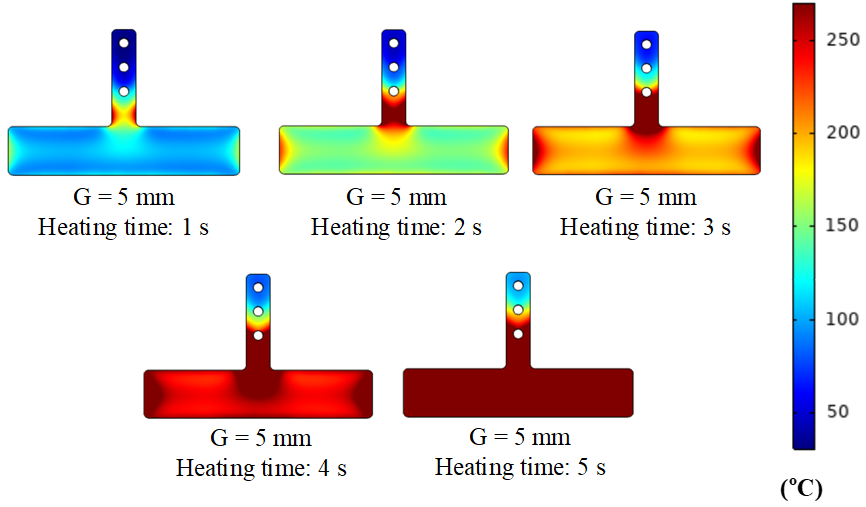

Supplement: S1 Appendix — Figure A1. Temperature distribution of Insert at the heating time from 1 s to 5 s with the gap (G) of 5 mm. This is the simulation results. Figure A2. Temperature distribution of Insert at the heating time from 1 s to 5 s with the gap (G) of 10 mm. This is the simulation results. Figure A3. Temperature distribution of Insert at the heating time from 1 s to 5 s with the gap (G) of 15 mm. This is the simulation results. Figure A4. Temperature distribution of a cavity plate with different heating times under a gap (G) of 5 mm. This is the experiment results. Figure A5. Temperature distribution of a cavity plate with different heating times under a gap (G) of 10 mm. This is the experiment results. Figure A6. Temperature distribution of a cavity plate with different heating times under a gap (G) of 15 mm. This is the experiment results. (ZIP) [file pone.0337889.s001.zip › Figure A1.png]

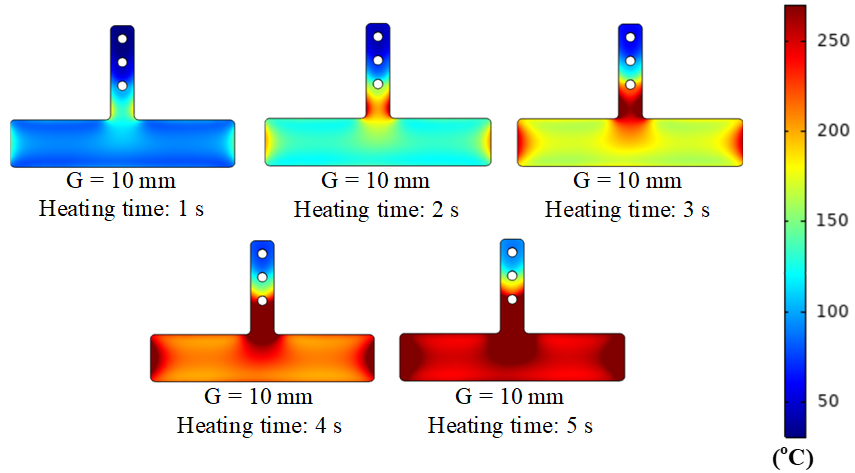

Supplement: S1 Appendix — Figure A1. Temperature distribution of Insert at the heating time from 1 s to 5 s with the gap (G) of 5 mm. This is the simulation results. Figure A2. Temperature distribution of Insert at the heating time from 1 s to 5 s with the gap (G) of 10 mm. This is the simulation results. Figure A3. Temperature distribution of Insert at the heating time from 1 s to 5 s with the gap (G) of 15 mm. This is the simulation results. Figure A4. Temperature distribution of a cavity plate with different heating times under a gap (G) of 5 mm. This is the experiment results. Figure A5. Temperature distribution of a cavity plate with different heating times under a gap (G) of 10 mm. This is the experiment results. Figure A6. Temperature distribution of a cavity plate with different heating times under a gap (G) of 15 mm. This is the experiment results. (ZIP) [file pone.0337889.s001.zip › Figure A2.png]

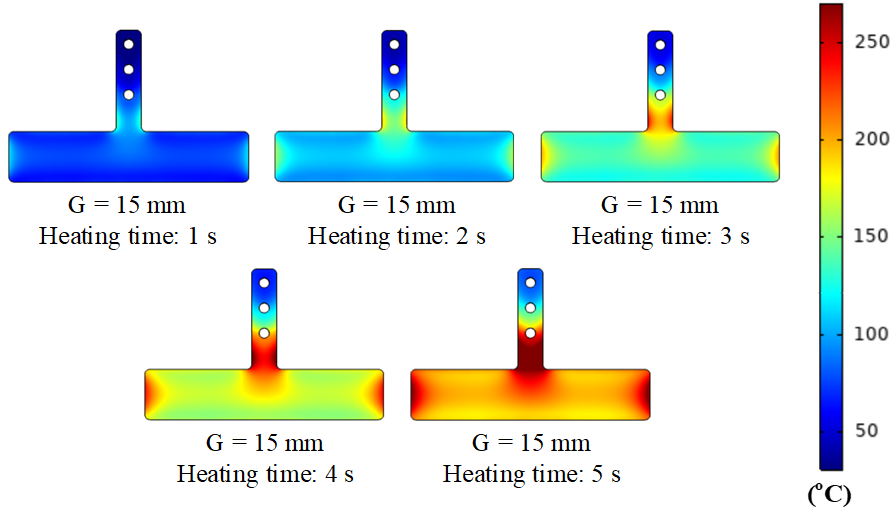

Supplement: S1 Appendix — Figure A1. Temperature distribution of Insert at the heating time from 1 s to 5 s with the gap (G) of 5 mm. This is the simulation results. Figure A2. Temperature distribution of Insert at the heating time from 1 s to 5 s with the gap (G) of 10 mm. This is the simulation results. Figure A3. Temperature distribution of Insert at the heating time from 1 s to 5 s with the gap (G) of 15 mm. This is the simulation results. Figure A4. Temperature distribution of a cavity plate with different heating times under a gap (G) of 5 mm. This is the experiment results. Figure A5. Temperature distribution of a cavity plate with different heating times under a gap (G) of 10 mm. This is the experiment results. Figure A6. Temperature distribution of a cavity plate with different heating times under a gap (G) of 15 mm. This is the experiment results. (ZIP) [file pone.0337889.s001.zip › Figure A3.png]

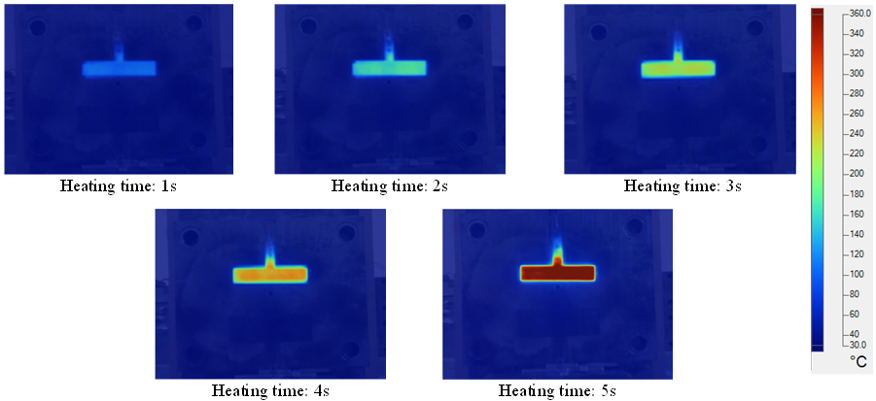

Supplement: S1 Appendix — Figure A1. Temperature distribution of Insert at the heating time from 1 s to 5 s with the gap (G) of 5 mm. This is the simulation results. Figure A2. Temperature distribution of Insert at the heating time from 1 s to 5 s with the gap (G) of 10 mm. This is the simulation results. Figure A3. Temperature distribution of Insert at the heating time from 1 s to 5 s with the gap (G) of 15 mm. This is the simulation results. Figure A4. Temperature distribution of a cavity plate with different heating times under a gap (G) of 5 mm. This is the experiment results. Figure A5. Temperature distribution of a cavity plate with different heating times under a gap (G) of 10 mm. This is the experiment results. Figure A6. Temperature distribution of a cavity plate with different heating times under a gap (G) of 15 mm. This is the experiment results. (ZIP) [file pone.0337889.s001.zip › Figure A4.png]

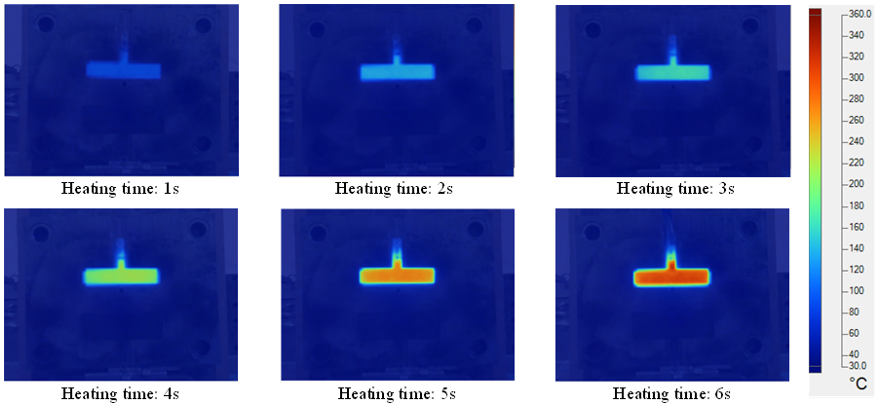

Supplement: S1 Appendix — Figure A1. Temperature distribution of Insert at the heating time from 1 s to 5 s with the gap (G) of 5 mm. This is the simulation results. Figure A2. Temperature distribution of Insert at the heating time from 1 s to 5 s with the gap (G) of 10 mm. This is the simulation results. Figure A3. Temperature distribution of Insert at the heating time from 1 s to 5 s with the gap (G) of 15 mm. This is the simulation results. Figure A4. Temperature distribution of a cavity plate with different heating times under a gap (G) of 5 mm. This is the experiment results. Figure A5. Temperature distribution of a cavity plate with different heating times under a gap (G) of 10 mm. This is the experiment results. Figure A6. Temperature distribution of a cavity plate with different heating times under a gap (G) of 15 mm. This is the experiment results. (ZIP) [file pone.0337889.s001.zip › Figure A5.png]
