# Supplementary material for: Applying a new mold temperature control strategy to improve the tensile strength of thin wall products in injection molding processes
Source: PLoS One. 2025 Dec 5;20(12):e0337889. doi: 10.1371/journal.pone.0337889 (PMC12680320; doi:10.1371/journal.pone.0337889)
Supplement: Attachment — Submitted filename: PONE-D-25-07599_review report.pdf [file pone.0337889.s002.pdf]

Dear Editor and Author:

Thank you for inviting me to review this exciting manuscript.

This study simulated and experimentally investigated the heating process using Ex-IHRSC. The temperature distribution of the insert was investigated by varying the distance between the induction coil and the mold surface (G = 5, 10, and 15 mm) and the heating time (1-8 s). In addition, The effect of the heating time on the tensile strengths of PA6, PP, PC, and ABS was investigated. This emphasizes the practical application of the research. However, the manuscript currently contains numerous errors that need to be fixed. By correcting these issues, the authors can significantly enhance the quality and impact of the work, making it a valuable contribution to the field. Here are the following specific comments and suggestions.

- Thoroughly check the manuscript for all spelling and grammar errors.
- The abbreviation is incorrect. For example, gas heating (GMTC),
- The abstract should include the primary purpose of the manuscript and a description of the research conducted.
- The historical contributions of prior research are not well-defined in the Introduction. The introduction section should include the primary purpose of the manuscript.
- In section 3, the study should discuss and compare the research findings with those of other research
- In section 4. Conclusions include the most important results from the study
- Add the measuring equipment in section 2 (thermal sensor, infrared camera, tensile test machine, etc).
- Material parameters and molding process parameters need to be shown in the manuscript.
- The tensile testing standard has not been clarified.
- The term “Ex-IHRSC” is not explained at its first appearance.
- Figures 11 and 19 need to show error bars (deviation).
- Figure 10 has no citation. Figure 18 is unclear, and the scale of each figure needs to be the same.
- Rows 367-368 and 630-633 are unclear.
- Many formulas are given in the introduction but are not used in the results and discussion section to explain the results.
- Why does longer heating time lead to higher strength in PA6 material?
- The material structure at the weld line needs to be checked.
- Add more recent references (within 5 years from 2025)

Thank you for reading.
