# Supplementary material for: Applying a new mold temperature control strategy to improve the tensile strength of thin wall products in injection molding processes
Source: PLoS One. 2025 Dec 5;20(12):e0337889. doi: 10.1371/journal.pone.0337889 (PMC12680320; doi:10.1371/journal.pone.0337889)
Supplement: Attachment — Submitted filename: PONE-D-25-07599_FDS.pdf [file pone.0337889.s003.pdf]

PLOS ONE

Applying a new mold temperature control strategy to reduce the cycle time and improve the tensile strength of thin wall products in injection molding processes

--Manuscript Draft--

**Manuscript Number:**	PONE-D-25-07599
**Article Type:**	Research Article
**Full Title:**	Applying a new mold temperature control strategy to reduce the cycle time and improve the tensile strength of thin wall products in injection molding processes
**Short Title:**	Optimized Mold Temperature Control for Faster Cycles and Stronger Thin-Wall Injection Molded Products
**Corresponding Author:**	Duc Thuan Huynh, M.D.<br>Trường Đại học Bách khoa Đại học Quốc gia Thành phố Hồ Chí Minh: VNUHCM-Ho Chi Minh City University of Technology<br>Ho Chi Minh City, Select State/Province VIET NAM
**Keywords:**	Injection molding; cycle time; heating time; weld-line tensile strength; mold heating; dynamic mold temperature control; induction heating; thin wall product
**Abstract:**	Mold temperature control plays an important role in the injection molding process and directly influences both the product quality and production efficiency. A high mold temperature improves the surface quality of the molded parts but extends the cooling time and overall cycle duration. However, a lower mold temperature shortens the cycle but can lead to many problems, such as weak weld lines or incomplete filling. This study investigated a novel approach that involves preheating the mold insert using induction heating before placing it in the mold. This method aims to reduce or eliminate the heating cycle time while ensuring an optimal mold temperature at the moment of polymer injection. The results show that this approach enables significantly faster heating, compared with conventional methods, while maintaining a stable mold temperature without extending the molding cycle. The heating rate reached an optimal level within a few seconds, thereby reducing the waiting time of the molding process. Additionally, preheating the mold insert can improve the tensile strength of the molded product, particularly for materials such as PC, ABS, PA6, and PP. For example, without preheating, the tensile strength of the PC was only 111.9 MPa, whereas after 6 s of induction heating, it increased to 123 MPa before decreasing slightly when the heating duration was extended. Similarly, ABS achieved a maximum tensile strength of 91.3 MPa after 4 s of heating, whereas PA6 and PP also exhibited notable improvements, thus reinforcing the weld line integrity and overall mechanical performance. These results confirm that integrating induction heating for preheating mold inserts not only optimizes the production time but also significantly enhances the mechanical properties of the injection-molded components. This approach presents a promising advancement in mold temperature control, improving both the manufacturing efficiency and product quality.
**Order of Authors:**	Thanh Trung Do
	Duc Thuan Huynh
	Tran Anh Son
	Pham Son Minh
**Additional Information:**	
**Question**	**Response**
**Financial Disclosure**<br><br>Enter a financial disclosure statement that describes the sources of funding for the work included in this submission. Review the submission guidelines for detailed	Yes

Powered by Editorial Manager® and ProduXion Manager® from Aries Systems Corporation

requirements. View published research articles from *PLOS ONE* for specific examples.<br><br>This statement is required for submission and **will appear in the published article** if the submission is accepted. Please make sure it is accurate.<br><br>**Funded studies**<br>Enter a statement with the following details:<br>• Initials of the authors who received each award<br>• Grant numbers awarded to each author<br>• The full name of each funder<br>• URL of each funder website<br>• Did the sponsors or funders play any role in the study design, data collection and analysis, decision to publish, or preparation of the manuscript?<br><br>Did you receive funding for this work?	
Please add funding details.<br>as follow-up to "**Financial Disclosure**<br><br>Enter a financial disclosure statement that describes the sources of funding for the work included in this submission. Review the submission guidelines for detailed requirements. View published research articles from *PLOS ONE* for specific examples.<br><br>This statement is required for submission and **will appear in the published article** if the submission is accepted. Please make sure it is accurate.	This research was funded by the Ministry of Education and Training, project grant No. T2024-02NTĐ, and hosted by Ho Chi Minh City University of Technology and Education, Vietnam.

Powered by Editorial Manager® and ProduXion Manager® from Aries Systems Corporation

**Funded studies**<br>Enter a statement with the following details:<br>• Initials of the authors who received each award<br>• Grant numbers awarded to each author<br>• The full name of each funder<br>• URL of each funder website<br>• Did the sponsors or funders play any role in the study design, data collection and analysis, decision to publish, or preparation of the manuscript?<br><br>Did you receive funding for this work?"	
Please select the country of your main research funder (please select carefully as in some cases this is used in fee calculation).<br>as follow-up to "**Financial Disclosure**<br><br>Enter a financial disclosure statement that describes the sources of funding for the work included in this submission. Review the submission guidelines for detailed requirements. View published research articles from *PLOS ONE* for specific examples.<br><br>This statement is required for submission and **will appear in the published article** if the submission is accepted. Please make sure it is accurate.<br><br>**Funded studies**<br>Enter a statement with the following details:<br>• Initials of the authors who received each award<br>• Grant numbers awarded to each author<br>• The full name of each funder<br>• URL of each funder website<br>• Did the sponsors or funders play any role in the study design, data collection and analysis, decision to publish, or preparation of the manuscript?<br><br>Did you receive funding for this work?"	VIET NAM - VN
**Competing Interests**	The authors have declared that no competing interests exist.

Use the instructions below to enter a competing interest statement for this submission. On behalf of all authors, disclose any competing interests that could be perceived to bias this work—acknowledging all financial support and any other relevant financial or non-financial competing interests.<br><br>This statement is required for submission and **will appear in the published article** if the submission is accepted. Please make sure it is accurate and that any funding sources listed in your Funding Information later in the submission form are also declared in your Financial Disclosure statement.<br><br>View published research articles from *PLOS ONE* for specific examples.<br><br>**NO authors have competing interests**<br><br>Enter: *The authors have declared that no competing interests exist.*<br><br>**Authors with competing interests**<br><br>Enter competing interest details beginning with this statement:<br><br>*I have read the journal's policy and the authors of this manuscript have the following competing interests: [insert competing interests here]*<br><br>* typeset	
**Ethics Statement**<br><br>Enter an ethics statement for this submission. This statement is required if the study involved:<br><br>• Human participants<br>• Human specimens or tissue<br>• Vertebrate animals or cephalopods<br>• Vertebrate embryos or tissues	N/A

• Field research<br><br>Write "N/A" if the submission does not require an ethics statement.<br><br>General guidance is provided below. Consult the submission guidelines for detailed instructions. **Make sure that all information entered here is included in the Methods section of the manuscript.**	

Powered by Editorial Manager® and ProduXion Manager® from Aries Systems Corporation

**Format for specific study types**<br><br>**Human Subject Research (involving human participants and/or tissue)**<br>• Give the name of the institutional review board or ethics committee that approved the study<br>• Include the approval number and/or a statement indicating approval of this research<br>• Indicate the form of consent obtained (written/oral) or the reason that consent was not obtained (e.g. the data were analyzed anonymously)<br><br>**Animal Research (involving vertebrate animals, embryos or tissues)**<br>• Provide the name of the Institutional Animal Care and Use Committee (IACUC) or other relevant ethics board that reviewed the study protocol, and indicate whether they approved this research or granted a formal waiver of ethical approval<br>• Include an approval number if one was obtained<br>• If the study involved *non-human primates*, add *additional details* about animal welfare and steps taken to ameliorate suffering<br>• If anesthesia, euthanasia, or any kind of animal sacrifice is part of the study, include briefly which substances and/or methods were applied<br><br>**Field Research**<br><br>Include the following details if this study involves the collection of plant, animal, or other materials from a natural setting:<br>• Field permit number<br>• Name of the institution or relevant body that granted permission	
**Data Availability**<br><br>Authors are required to make all data underlying the findings described fully available, without restriction, and from the time of publication. PLOS allows rare exceptions to address legal and ethical concerns. See the PLOS Data Policy and FAQ for detailed information.	Yes - all data are fully available without restriction

A Data Availability Statement describing where the data can be found is required at submission. Your answers to this question constitute the Data Availability Statement and **will be published in the article**, if accepted.<br><br>**Important:** Stating 'data available on request from the author' is not sufficient. If your data are only available upon request, select 'No' for the first question and explain your exceptional situation in the text box.<br><br>Do the authors confirm that all data underlying the findings described in their manuscript are fully available without restriction?	
**Describe where the data may be found in full sentences. If you are copying our sample text, replace any instances of XXX with the appropriate details.**<br><br>• If the data are **held or will be held in a public repository**, include URLs, accession numbers or DOIs. If this information will only be available after acceptance, indicate this by ticking the box below. For example: *All XXX files are available from the XXX database (accession number(s) XXX, XXX.).*<br>• If the data are all contained **within the manuscript and/or Supporting Information files**, enter the following: *All relevant data are within the manuscript and its Supporting Information files.*<br>• If neither of these applies but you are able to provide **details of access elsewhere**, with or without limitations, please do so. For example:<br><br>*Data cannot be shared publicly because of [XXX]. Data are available from the XXX Institutional Data Access / Ethics Committee (contact via XXX) for researchers who meet the criteria for access to confidential data.*<br><br>*The data underlying the results presented in the study are available from (include the name of the third party*	All relevant data are within the manuscript and its Supporting Information files.

Powered by Editorial Manager® and ProduXion Manager® from Aries Systems Corporation

and contact information or URL).<br>• This text is appropriate if the data are owned by a third party and authors do not have permission to share the data.<br><br>* typeset	
Additional data availability information:	

Manuscript

Click here to access/download;Manuscript;Paper_Submit PLOS ONE.docx

Applying a new mold temperature control strategy to reduce the cycle time and improve the tensile strength of thin wall products in injection molding processes

Thanh Trung Do[1, a], Duc Thuan Huynh[2,3,4,b *], Tran Anh Son[2,3,c], and Pham Son Minh[1,d]

[1]University of Technology and Education, Ho Chi Minh City, Vietnam

[2]Ho Chi Minh City University of Technology (HCMUT), 700000, Vietnam

[3]Vietnam National University-Ho Chi Minh City (VNU-HCM), 700000, Vietnam

[4]Tran Dai Nghia University (TDNU), 700000, Vietnam

[a] trungdt@hcmute.edu.vn, [b] hdthuan.sdh241@hcmut.edu.vn, [c]tason@hcmut.edu.vn,

[d]minhps@hcmute.edu.vn

Abstract

Mold temperature control plays an important role in the injection molding process and directly influences both the product quality and production efficiency. A high mold temperature improves the surface quality of the molded parts but extends the cooling time and overall cycle duration. However, a lower mold temperature shortens the cycle but can lead to many problems, such as weak weld lines or incomplete filling. This study investigated a novel approach that involves preheating the mold insert using induction heating before placing it in the mold. This method aims to reduce or eliminate the heating cycle time while ensuring an optimal mold temperature at the moment of polymer injection. The results show that this approach enables significantly faster heating, compared with conventional methods, while maintaining a stable mold temperature without extending the molding cycle. The heating rate reached an optimal level within a few seconds, thereby reducing the waiting time of the molding process. Additionally, preheating the mold insert can improve the tensile strength of the molded product, particularly for materials such as PC, ABS, PA6, and PP. For example, without preheating, the tensile strength of the PC was only 111.9 MPa, whereas after 6 s of induction heating, it increased to 123 MPa before decreasing slightly when the heating duration was extended. Similarly, ABS achieved a maximum tensile strength of 91.3 MPa after 4 s of heating, whereas PA6 and PP also exhibited notable improvements, thus reinforcing the weld line integrity and overall mechanical performance. These results confirm that integrating induction heating for preheating mold inserts not only optimizes the production time but also significantly enhances the mechanical properties of the injection-molded components. This approach presents a promising advancement in mold temperature control, improving both the manufacturing efficiency and product quality.

Keywords: Injection molding, cycle time, heating time, weld-line tensile strength, mold heating, dynamic mold temperature control, induction heating, thin wall product

I. Introduction

Injection molding is becoming increasingly prevalent and plays a crucial role in the manufacturing of plastic and composite components. In injection molding, mold temperature control is essential in ensuring product quality and optimizing the production cycle [1–5]. A high mold surface temperature improves the surface finish of the molded parts, facilitates the filling of molten plastic, and reduces defects, such as weak weld lines or incomplete filling. However, maintaining a high mold temperature prolongs the cooling phase, leading to an increase in the overall molding cycle time. However, lowering the mold temperature shortens the cooling time but may compromise surface quality and exacerbate flow-related issues. Therefore, a key challenge in modern injection molding technology is to effectively elevate the mold temperature without negatively impacting the production efficiency. Therefore, this study focuses on addressing this issue by developing an advanced induction heating process for mold temperature control with the aim of achieving rapid heating while maintaining an optimal balance between quality and cycle time.

In recent years, thin-wall injection molding and microinjection molding have become essential technologies in the manufacturing of polymer components, particularly in the fields of microoptics [6,7] and microfluidics [8,9]. Applications such as optical lenses, optical switches [10,11], waveguides [12,13], and lab-on-a-chip devices [14,15] require precise mold temperature control to ensure high dimensional accuracy and product performance. Additionally, advanced

cooling strategies, such as conformal cooling channels, have been developed to reduce cooling time without compromising product quality.

Various heating techniques have been investigated and implemented to achieve flexible mold temperature control. Among these, dynamic mold temperature control (DMTC) has emerged as an important approach, as it allows for rapid mold heating during the filling phase followed by immediate cooling [16–18]. The primary objective of DMTC is to eliminate the frozen layer that forms on the mold surface during injection molding, thereby improving the melt flow behavior and enhancing the mechanical properties of the molded products. This study focuses on optimizing this process using induction heating, which enables rapid and localized temperature adjustments and improves molding efficiency while maintaining high product quality.

Mold heating techniques have been extensively studied to optimize performance and ensure product quality in injection molding processes. A common method involves using hot water at temperatures between 90 and 100 °C to preheat the mold before injection. This method is slow and thus prevents the mold surface from reaching the desired temperature in a short time, particularly in applications requiring high mold temperatures. Additionally, resistance heating is widely used, owing to its simple design and ease of use [19–23]. However, the temperature increase achieved using this method is typically limited to a few tens of degrees Celsius above the initial temperature, making it less effective for applications that require rapid and high-temperature heating. Another approach is steam heating, which can achieve higher mold temperatures than can the two aforementioned methods [24]. However, the implementation of steam heating requires a complex temperature control system, which significantly increases both the investment and operational costs and thus limits its feasibility for use in large-scale manufacturing. These

limitations highlight the urgent need for more advanced mold heating technologies that can rapidly increase the mold temperature while ensuring precise thermal distribution control without significantly extending the production cycle.

Currently, various advanced heating technologies have been researched and developed to optimize DMTC and enhance the quality of injection-molded products without significantly increasing the production cycle time. Among these, methods such as induction heating [25–28], infrared heating [29–32], and gas-assisted mold temperature control (GMTC) [33–36] are promising. The GMTC method operates by directing hot gas flow onto the mold surface, which increases its temperature to thereby ensure a uniform heat distribution and reduce the formation of the frozen layer in the polymer melt. This helps to improve the surface and enhance the mold filling performance. However, the GMTC entails a high cost of operation, particularly when maintaining stable hot gas temperatures over an extended period. In contrast, induction heating provides several advantages by utilizing electromagnetic fields to generate eddy currents within the mold, thereby enabling rapid heating without direct contact with the mold surface. This method reduces the heat loss and provides greater flexibility, making it suitable for molds with complex geometries. Additionally, induction heating has high energy efficiency and consumes less power than do traditional heating methods such as resistance or steam heating.

Despite these benefits, one of the biggest problems associated with modern mold heating techniques is the potential increase in the injection molding cycle time owing to the additional heating phase before polymer injection. This may affect the production efficiency, especially in high-volume manufacturing environments. Consequently, research efforts have focused on optimizing heating strategies to effectively integrate heating time within the molding cycle and

thus prevent unnecessary delays while maintaining superior product quality. Developing a heating process that maximizes efficiency without extending the cycle time remains a critical objective in current injection molding research.

To overcome the limitations of traditional mold heating methods, this study proposes an approach that preheats the mold insert during the injection molding process rather than heating the entire mold cavity. During the injection process, a separate insert was independently heated. Once the previously molded product was ejected, the preheated insert, which had already reached the desired temperature, was immediately placed in the mold. This method offers several significant advantages, particularly in terms of reducing the overall cycle time. Because the heating process occurs simultaneously with the injection phase, unnecessary waiting time prior to the polymer injection is eliminated. When the preheated insert was placed in the mold, the mold temperature instantly reached an optimal level, which significantly improved the flowability of the molten polymer. Consequently, the filling process was accelerated, which minimized defects, such as weak weld lines and incomplete filling. This approach enhances production efficiency without compromising product quality. This method optimizes the overall performance of the injection molding process by significantly reducing the heating time before polymer injection. This is particularly beneficial for applications requiring high-speed production and precise dimensional accuracy, making it a promising solution for modern injection molding technologies.

This study integrated numerical simulations and experimental validation to assess the effectiveness of the mold insert heating approach in injection molding. Thermal simulations using COMSOL Multiphysics were conducted to analyze the temperature distribution on the insert and evaluate the impact of the heating process on the mold cavity, thus ensuring optimal temperature

maintenance throughout the production cycle. Additionally, polymer flow simulations using Moldex3D were employed to examine the mold filling capability, influence of mold temperature on polymer flow behavior, and potential formation of defects, such as weak weld lines or incomplete filling. To validate the accuracy of the simulations, real-time temperature measurements were performed using thermal sensors and infrared cameras to allow for a direct comparison with the simulation data. Finally, the mechanical properties of the injection-molded products were assessed via tensile strength testing, which enabled a comparative analysis between the proposed heating method and conventional mold heating techniques. The findings of this study will help determine the feasibility of implementing this method in industrial production to optimize both efficiency and product quality in modern injection molding applications.

2. Simulation and experimental methods

2.1. Principle of induction heating

The simulation of induction heating is a coupled problem involving both electromagnetic induction and heat transfer. In this study, a two-step method was used to efficiently reduce the computational time and memory usage.

In the first step, the electromagnetic problem was solved to determine the eddy current and hysteresis losses. However, according to a study by Rudnev et al. [37], the hysteresis loss accounts for only approximately 7% of the total energy loss, compared with the eddy current loss in most induction heating applications. Therefore, it is often neglected in simulations [37]. The results of the electromagnetic simulation are used as a heat source to solve the thermal conduction problem.

Induction heating follows Maxwell's equations, which describe the propagation of magnetic fields and electric currents in conductive materials:

$$\nabla \times H = J + \frac{\partial D}{\partial t} \quad (1)$$

$$\nabla \times E = -\frac{\partial B}{\partial t} \quad (2)$$

$$\nabla . B = 0 \quad (3)$$

$$\nabla . D = \rho \quad (4)$$

where:

- H is the magnetic field intensity,
- J is the current density,
- D is the electric flux density,
- E is the electric field intensity,
- B is the magnetic flux density,
- and ρ is the charge density.

The relationships between these physical parameters are given by:

$$D = \varepsilon_0 \varepsilon_r E \quad (5)$$

$$B = \mu_0 \mu_r H \quad (6)$$

$$J = \sigma E \quad (7)$$

where σ is the electrical conductivity, μ is the magnetic permeability, and ε is the dielectric permittivity of the material.

Previous studies have shown that the efficiency of induction heating can be optimized by adjusting the frequency of the alternating current and the gap between the induction coil and heated material [38].

To calculate the temperature distribution induced by induction heating, the transient heat conduction equation was solved as follows:

$$\rho C_p \frac{\partial T}{\partial t} - \nabla.(k \nabla T) = Q \quad (8)$$

where:

- T is the temperature,
- ρ is the material density,
- C_p is the specific heat capacity,
- k is the thermal conductivity,
- and Q represents the heat source generated by eddy currents.

By solving these coupled electromagnetic and thermal equations, the heating performance of induction systems can be accurately analyzed to enable the optimization of the mold temperature control in the injection molding process.

2.2. Application of induction heating to increase the cavity temperature

External induction heating with rotational structure for mold temperature control (Ex-IHRSC) is an advanced technique designed to efficiently regulate mold temperature during the injection molding process. This method enables the direct and rapid heating of the mold cavity surface, thus optimizing the heat distribution without significantly increasing the overall mold temperature. The operating mechanisms of the Ex-IHRSC are based on the principle of electromagnetic induction, which concentrates energy on the mold surface to ensure a stable temperature before the molten polymer fills the cavity.

The system consists of two main components: an induction heating unit and a rotational structure that exchanges the mold inserts. The induction heating unit can reach temperatures of up to 320 °C, thereby allowing the mold surface to quickly attain the desired temperature. This rapid heating improves the flowability of the molten plastic, enhances mold filling, and improves the surface quality. Simultaneously, a precisely controlled cooling system ensured that the mold temperature was quickly reduced to the optimal level immediately after the injection molding cycle. In addition, temperature sensors were integrated into key locations within the mold to provide real-time temperature data. This ensures accurate heat and cooling control and minimizes defects such as weak welding lines, incomplete filling, and inaccuracy in dimensions caused by uncontrolled temperature fluctuations.

Fig 1 illustrates the induction heating process applied to the injection mold, which aims to increase the surface temperature of the mold immediately before the molten polymer enters the cavity. This system uses an induction coil to generate thermal energy via electromagnetic induction, thereby enabling rapid and localized heating without direct contact. The process was

performed in five main steps to ensure efficient mold temperature control without extending the overall cycle time.

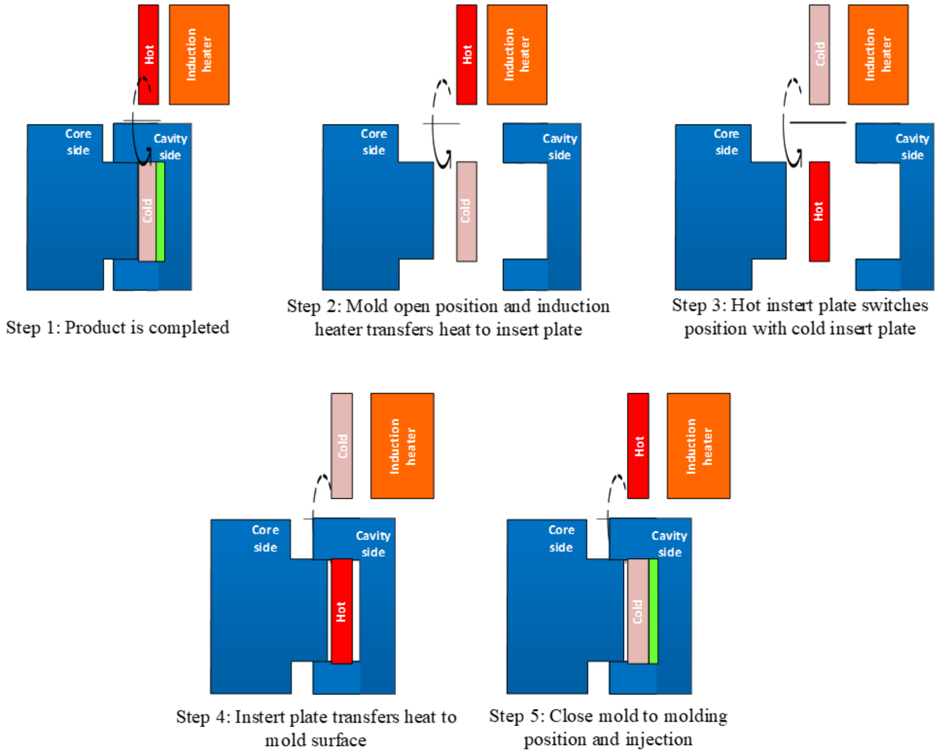

Fig 1. Mold position in the heating stage of the Ex-IHRSC process

In the first step, the completed injection-molded product was ejected from the mold, and the mold was opened to prepare for the heating phase. This mold opening allowed the induction heating system to access the surface of the mold insert directly to ensure optimal heat transfer efficiency. In step two, the two halves of the mold move to a fully open position, which creates space for the induction coil to operate. As a high-frequency alternating current flows through the coil, a variable magnetic field is generated. This magnetic field interacts with the mold insert, which is made of an electrically conductive material. This interaction induces eddy currents within the insert, which rapidly heat its surface via Joule heating. This method provides fast and

uniform heating without direct contact, making it superior to conventional heating techniques, such as resistance heating or infrared heating.

Step three involves the replacement of the mold insert. After the preheated insert reaches the desired temperature, it is placed in the main mold cavity, and a cooler insert is swapped in to prepare for the next cycle. The rotational mechanism ensures a stable mold temperature and the precise control of thermal variations within the mold. In step four, the heated mold insert increases the cavity surface temperature to its optimal level, thereby reducing the viscosity of the molten polymer, improving mold filling, and minimizing weld line defects. Once the desired temperature is reached, the mold is closed to initiate the injection-molding cycle in step five. In summary, an induction heating system combined with a rotational mold insert mechanism offers an efficient method for mold temperature control in injection molding. With rapid and precise heating that does not extend the cycle time, this approach has significant potential for application in industrial production, particularly for high-precision applications that require strict surface quality control.

Figs 2 and 3 illustrate the experimental setup for an external induction heating with rotational structure for mold temperature control (Ex-IHRSC) system. This system consists of several key components, including an induction heater, a mold insert plate, an induction coil, a rotating device, and a mold temperature controller. Each component plays a crucial role in regulating the mold temperature to ensure high efficiency in the injection molding process while improving product quality.

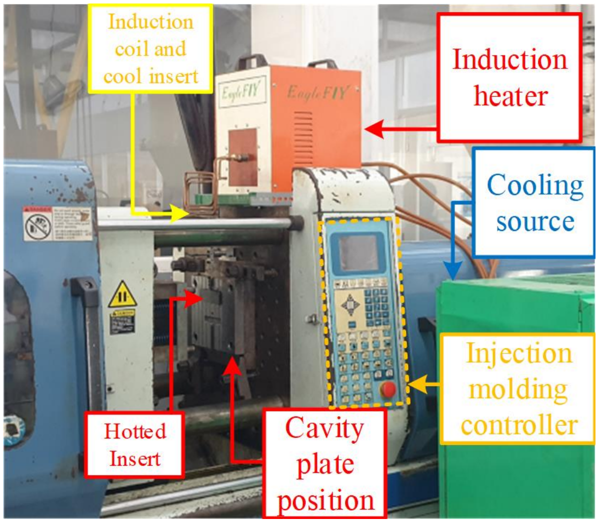

Fig 2. Ex-IHRSC experiment model

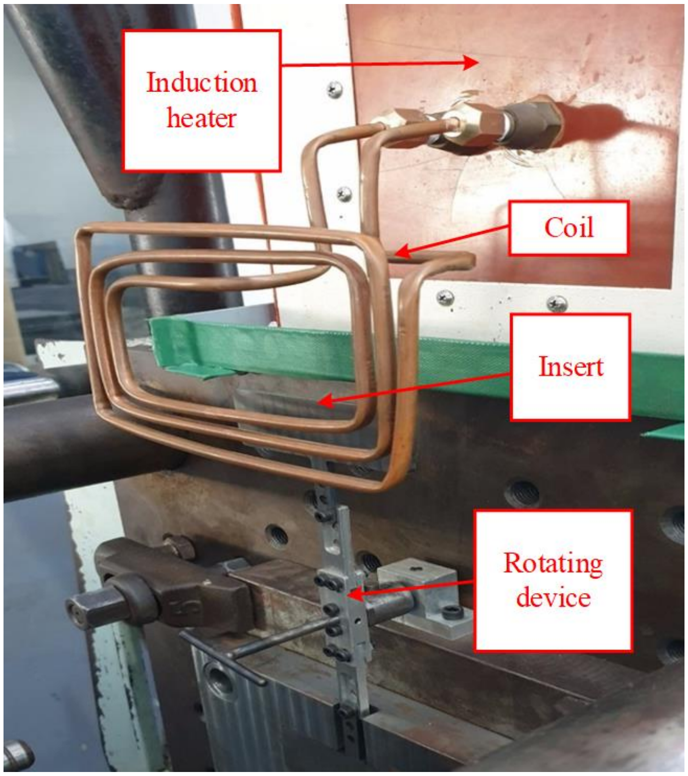

Fig 3. Hot gas generator

Fig 2 presents an overview of the layout of the Ex-IHRSC system in the experimental setup. The induction heater serves as the core component and operates by generating a high-frequency alternating current in the induction coil, which, in turn, produces variability in the magnetic field around the coil. When a mold insert made of an electrically conductive material is placed in this magnetic field, eddy currents are induced within the insert to rapidly heat its surface via Joule heating. This mechanism enables the rapid surface heating of the mold cavity without increasing the overall mold temperature, thereby optimizing both energy efficiency and cycle time.

In addition to the induction heater, the induction coil also plays a critical role in generating an alternating magnetic field. Fig 3 provides a more detailed view of the induction coil and how it surrounds the mold insert to optimize heat transfer. The gap distance (G) between the coil and mold surface was carefully controlled to achieve the highest heating efficiency while avoiding overheating or the occurence of an uneven temperature distribution.

A key feature of this system is its rotating device, which allows for the interchangeability of hot and cold mold inserts. This rotation mechanism ensures that while one insert is actively used in the injection molding process, the other insert is simultaneously preheated by the induction heating system. This approach allows for continuous molding cycles without interrupting production, effectively eliminating the in-cycle heating time and enhancing the overall manufacturing efficiency.

Fig 4 illustrates the locations of the temperature measurement points on the mold, including S1, S2, and S3, which are strategically positioned to monitor the temperature distribution during the heating and polymer filling process. The S2 point is located at the center

of the thin-wall region, where precise temperature control is required to ensure complete mold filling without premature solidification. Moreover, S1 and S3 are positioned at locations where the molten polymer enters the thin-wall section, thus allowing for an assessment of temperature variations before and during the filling process. The temperature distribution at these points plays a crucial role in analyzing the flow behavior of the molten polymer, determining thermal uniformity, and optimizing the heating process to enhance product quality.

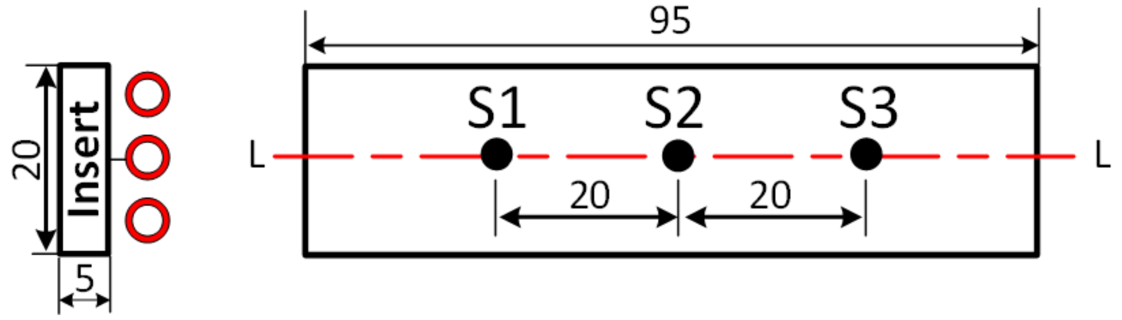

Fig 4. Cavity dimensions with the sensor locations S1 to S3

Fig 5 illustrates the heating locations in both the simulation and experimental setups, allowing for a direct comparison between the computed and measured data. In this study, a simulation model was configured to closely match the experimental conditions for analyzing the temperature distribution on the mold surface during induction heating.

The simulation images show that the high-temperature regions are concentrated in areas that are directly exposed to the induced magnetic field, particularly on the mold insert surface, where eddy currents generate heat. In the experimental setup, temperature sensors were placed at key measurement points (S1, S2, and S3) to collect real-time temperature variations. Additionally, a thermal camera was used to visually capture the temperature distribution in the mold to provide insights into the uniformity of the heating process.

The simulation results provide a detailed understanding of how heat propagates within the mold cavity, aiding in the optimization of parameters, such as the induction coil distance (G), input power, and heating duration. The deviation between the two approaches was evaluated by comparing the simulation and experimental data, and necessary adjustments were made to enhance model accuracy. This process not only optimizes the heating efficiency but also ensures that the mold reaches the desired temperature conditions before polymer injection, ultimately improving the product quality.

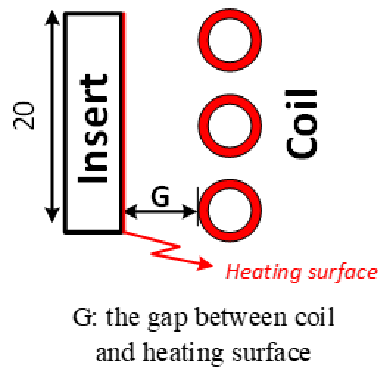

Fig 5. Heating position of Ex-GMTC

Fig 6 illustrates the meshing model applied in two key simulation stages: the heating and polymer-filling phases. The mesh process plays a crucial role in determining the accuracy of the simulation, as it directly affects the predicted temperature distribution within the mold and the flow behavior of the molten polymer.

To ensure a balance between the computational accuracy and processing time, the meshing model was optimized by employing appropriate meshing techniques tailored to the specific physical characteristics of the system. A finer mesh was applied in critical regions, such as the mold insert and polymer flow path, where precise thermal and flow analyses are essential.

Coarser meshing was used in less critical areas to improve computational efficiency. By refining the meshing strategy, the simulation effectively captured the heat transfer dynamics and polymer filling behavior, leading to more reliable predictions for optimizing the induction heating-assisted injection molding process.

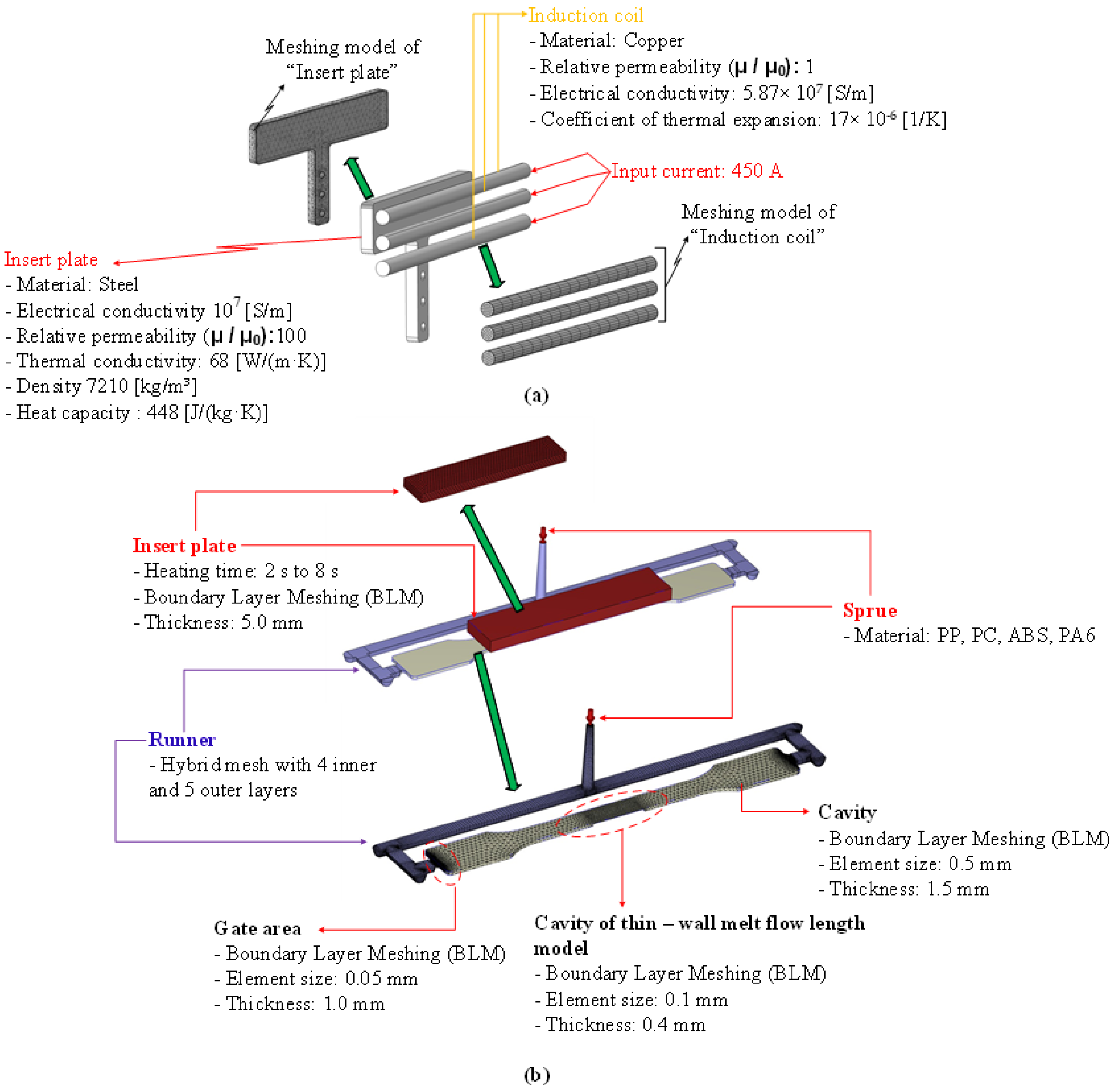

Fig 6. Meshing model of the heating (a) and polymer-filling (b) steps.

In the heating phase, a simulation was conducted to evaluate the heat transfer from the induction coil to the mold insert via the eddy current effect. When a high-frequency alternating current flows through the induction coil, a fluctuating magnetic field is generated. This magnetic field induces eddy currents within the mold insert. This phenomenon heats the mold surface via the Joule effect, with the highest temperature concentration occurring in the regions directly affected by the induced magnetic field.

The meshing structure shown in Fig 6a was designed to ensure a precise simulation of the heat transfer process. The induction coil modeling uses copper with an electrical conductivity of 5.87×107 S/m and a relative permeability of μ/μ0=1 to ensure high magnetic field generation and optimal heating efficiency. The mold insert is made of steel and has an electrical conductivity of 107 S/m and a relative permeability of μ/μ0 = 100, which optimizes its ability to absorb electromagnetic energy and generate uniform surface heating.

The mesh in the insert region was refined with high-density elements using boundary layer meshing (BLM) to accurately model the heat propagation. The boundary layer mesh thickness in this region was set to 5.0 mm, with heating durations ranging from 2 s to 8 s to ensure uniform heating across the mold surface. Moreover, the mesh size in the remaining mold regions was coarser to optimize the computational efficiency without significantly affecting the accuracy. Table 1 lists the additional simulation parameters. Once the mold reached the desired temperature, the polymer filling phase was simulated to analyze the flow behavior of the molten polymer and the effect of the mold temperature on the filling process. In this phase, the meshing model plays a crucial role in predicting phenomena such as weld line formation, air entrapment, and frozen layer development.

Table 1. Material properties (for simulation)

Material	Properties	Unit	Value
Air	Molecular mass	kg / kmol	28.96
	Density	kg/m3	1.185
	Specific heat capacity	J/kg °K	1004.4
	Dynamic viscosity	kg/ms	1.831e-5
	Thermal conductivity	W/m°K	0.0261
Steel	Molecular mass	kg/kmol	55.85
	Density	kg/m3	7854
	Specific heat capacity	J/kg °K	434
	Thermal conductivity	W/m°K	60.5

In Fig 6b, the meshing model is configured with smaller element sizes in the critical regions, particularly in the thin-walled section and gate area. The mesh in the gate area had an element size of 0.05 mm and a thickness of 1.0 mm, ensuring an accurate simulation of the temperature and pressure changes as the polymer entered the mold cavity. The cavity mesh had an element size of 0.5 mm and a thickness of 1.5 mm, providing high accuracy in flow behavior prediction. Moreover, the runner mesh adopted a hybrid mesh structure consisting of four inner

layers and five outer layers, thus optimizing the polymer filling process without excessively increasing the computational time.

The polymer materials used in the simulation, including PP, PC, ABS, and PA6, each with a different viscosity and thermal conductivity, affected the mold-filling process. The interface between the molten polymer and mold surface was modeled using BLM to accurately simulate the heat exchange between the polymer and mold, thus enabling precise predictions of the mold-filling capability and final product quality.Fig 7 shows the technical schema of the test specimen designed to evaluate the effect of the inductive heat technique on the polymer filling behavior and mechanical quality of injection-molded products. This test specimen was specially designed in accordance with tensile strength testing standards, with a reduced center area to simulate real industrial conditions, where a molten polymer is needed to fill thin-walled features. If the polymer does not fully fill the cavities or weld line defects, the tensile strength of the final product will affect its mechanical performance.

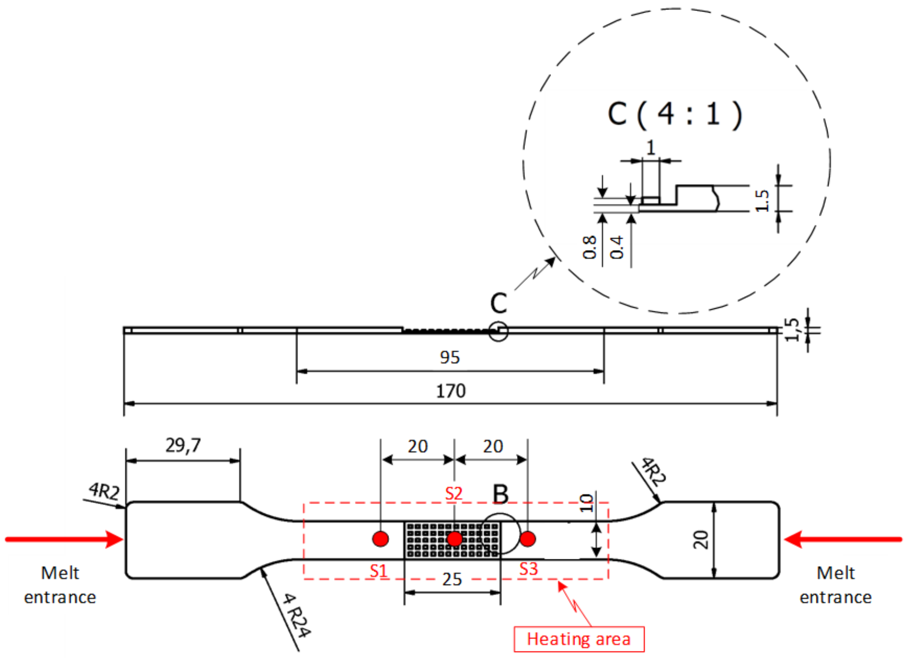

Fig 7. Tensile testing specimen dimensions.

The test specimen had a total length of 170 mm, with a maximum wideness of 29.7 mm in the largest area, narrowing to 10 mm in the central area where the heating process occurred. The thickness at the central area is 1.5 mm, with a special thin-wall section of only 0.8 mm, which allows for the study of how the polymer fills narrow cross-sections. The heating area is clearly shown with measurements of 25 × 20 mm and is the most important section in this study. During injection molding, the mold insert in this area is heated by induction, which increases the mold surface temperature before the polymer fills the cavity. The goals of this method are to reduce the number of frozen layers, improve the filling capacity, minimize weld line defects, and improve the quality of products.

Three temperature measurement points (S1, S2, and S3) were placed at important locations to monitor the temperature variation during the heating and polymer-filling phases. Point S2 was at the center of the heating area and was more critical than the others because the molten polymer directly contacted the mold cavity at its thin location (0.8 mm). Effective heating ensured that the polymer filled the mold without early solidification. Points S1 and S3 were placed 12.5 mm from the center and were used to observe the temperature of the polymer as it began to flow into the thin-walled section. If the temperature at these points is low, the polymer can solidify before the filling is completed, resulting in problems such as short shots or weak welding lines.

The flow directionality of the polymer during injection molding is clearly illustrated, showing how the molten polymer flows from both ends of the specimen toward the central heating area following the melt entrance into the heat zone. This setup was used to evaluate the

effect of mold temperature on polymer flowability. If the mold temperature is not sufficiently high, then the polymer may solidify before completely filling the central part, resulting in missing materials. In contrast, when the mold temperature is properly controlled, the polymer flow becomes smoother and more effective, thus enabling complete cavity filling and improving the surface quality of the finished product.

3. Results and discussion

3.1. Heating efficiency

The Ex-IHRSC models were designed for the fastest localized heat and to ensure an equal temperature distribution on the mold surface without increasing the overall mold temperature. This system is composed of an outer induction coil, a rotational structure for quick switching between hot and cold mold plates, and a real-time sensor.

This study evaluated the effect of the distance between the induction coil and the mold surface (G = 5, 10, and 15 mm) along with the heating time (1–8 s). The results presented in Figs 8, 9, and 10 indicate that the smallest distance enhanced the heating efficiency. When G = 5 mm, the mold surface temperature reached 300 °C in 5 s, whereas for G = 10 mm and 15 mm, the required time increased to 6 and 8 s, respectively. These results demonstrate that a stronger eddy current effect occurs at smaller distances, which allows for more efficient heat concentration.

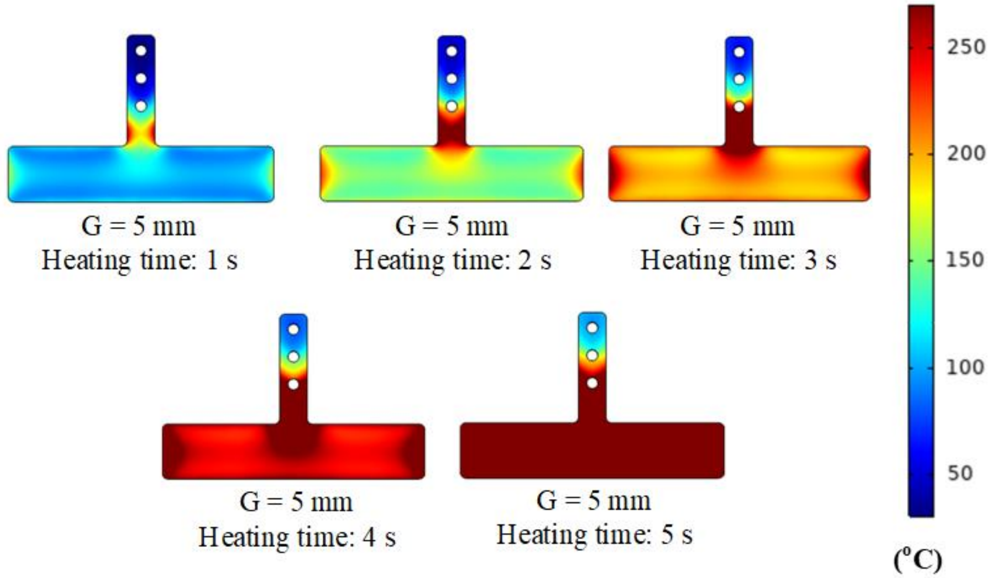

Fig 8: Temperature distribution of the insert at heating times from 1 s to 5 s with a gap (G) of 5 mm

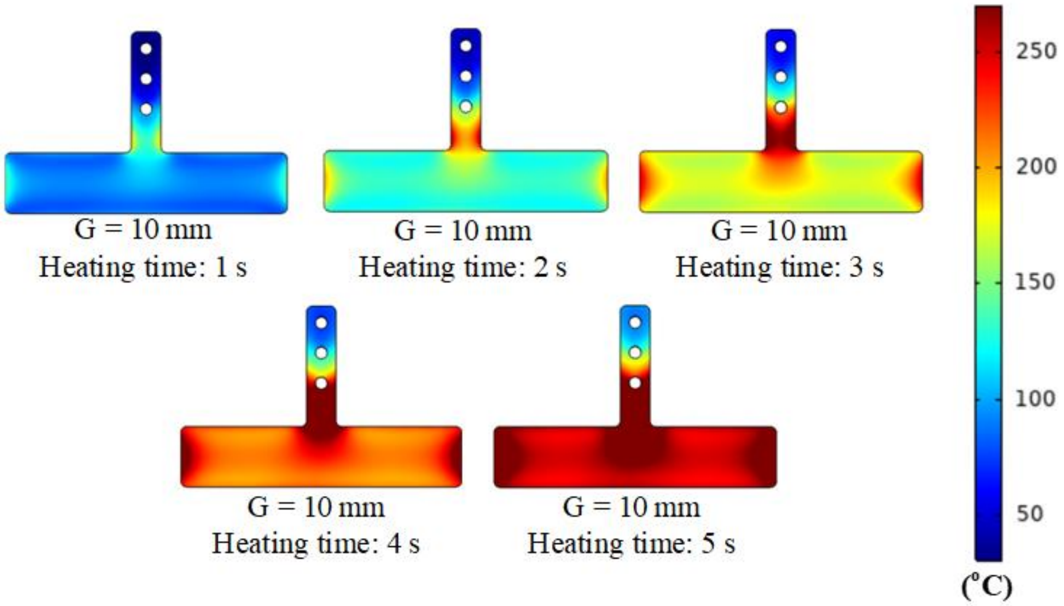

Fig 9: Temperature distribution of the insert at heating times from 1 s to 5 s with a gap (G) of 10 mm

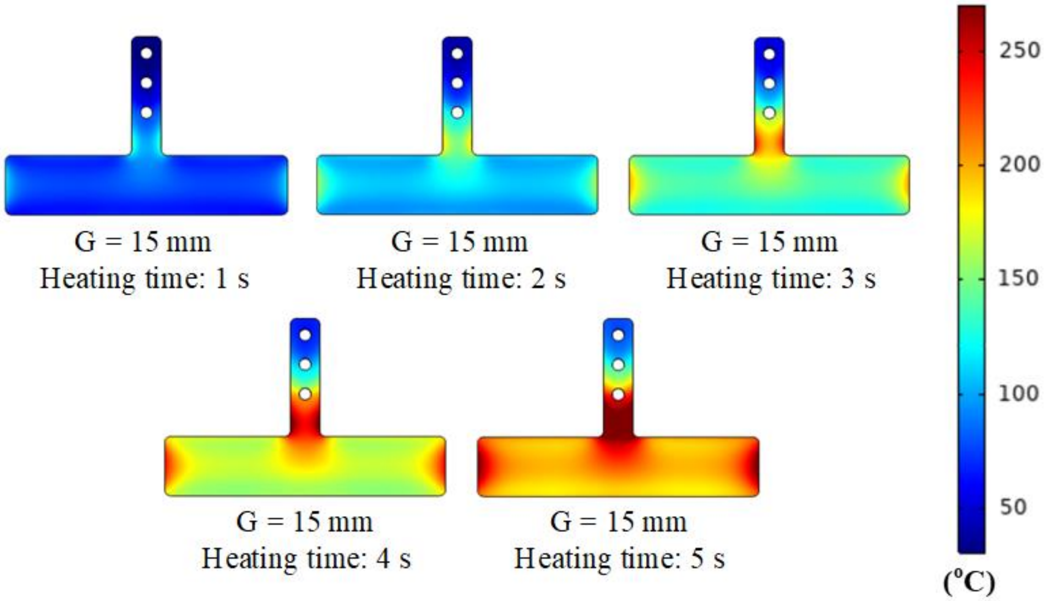

Fig 10: Temperature distribution of the insert at heating times from 1 s to 5 s with a gap (G) of 15 mm

Nevertheless, when the gaps are too small, the risk of overheating or contact between the coil and mold increases. A distance of G = 10 mm was considered optimal because it ensured highly efficient heating while maintaining the safety of the system. At G = 15 mm, the heating time was extended, but the risk of localized overheating was minimized, making it suitable for applications requiring high stability. One interesting issue is the edge effect, for which the temperature is more concentrated at the mold edges owing to stronger eddy currents in these areas. This effect can be controlled by optimizing the coil positioning or adjusting the heating parameters. Unlike traditional heating methods, which typically occur after the injection molding cycle ends, the Ex-IHRSC method integrates heating in a parallel manner with the injection cycle, thereby preventing any increase in production cycle time. Given that typical injection molding cycles range between 10 and 30 s, a heating duration of 5–8 s is reasonable. Overall, the Ex-IHRSC not only overcomes the limitations of existing heating methods but also significantly

enhances the heating efficiency and improves the quality of injection-molded products without decreasing the production efficiency.

3.2. Simulation and experiment comparison

The simulation process was conducted using the COMSOL Multiphysics software suite, which employed an electromagnetic-thermal module to model the temperature distribution within the mold under the impact of induction heating. The key input parameters included the distance between the induction coil and the mold surface (G = 5, 10, and 15 mm), thehe ating time (1–8 s), and a simulated mold design that matched the actual design with well-defined measurement positions at S1, S2, and S3. This simulation allowed for the prediction of the temperature distribution over the mold surface, particularly in areas that were susceptible to the edge effect.

An experiment was conducted on the Ex-IHRSC system using the same parameters as those used in the simulations. The temperatures at measurement points S1 (before the molten polymer enters the thin-wall region), S2 (central region), and S3 (before the molten polymer enters the thin-wall region) were recorded using an infrared camera that directly captured the mold surface temperature. In addition, a thermal camera was used to monitor the temperature distribution across the entire mold surface, facilitating a direct comparison with the simulation results. This process ensures consistency between the simulation and experimental conditions as well as provides a reliable foundation for assessing the accuracy of the simulation model.

(*) Temperature analysis at measurement points

Temperature at S1 and S3 (before the molten polymer enters the thin-wall region): Both the simulated and experimental results indicate that the temperatures at S1 and S3 increased more slowly than did that of S2, with the measured values ranging from 270 °C to 280 °C after 5 s (Fig 11). This is because S1 and S3 were located farther from the mold edges, where the edge effect was weaker, resulting in lower eddy current concentrations at these positions. Although the temperatures at S1 and S3 increased slowly, they remained relatively uniform between the two points, with a temperature deviation of no higher than 5 °C being observed in either the simulation or the experiment (Fig 12). Maintaining uniform heating at S1 and S3 helps reduce the frozen layer phenomenon that occurs when the polymer solidifies prematurely at the mold edges. This improvement in temperature distribution facilitates better polymer flow into the mold cavity, which is critical to ensure full filling, particularly for complicated and high-precision molded products.

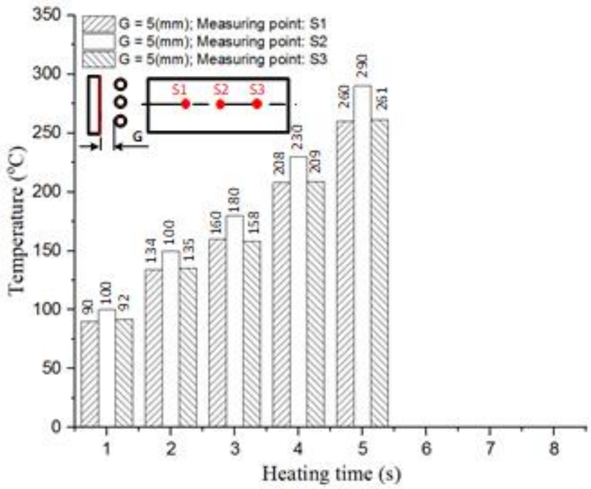

(a) The gap (G) of 5 mm

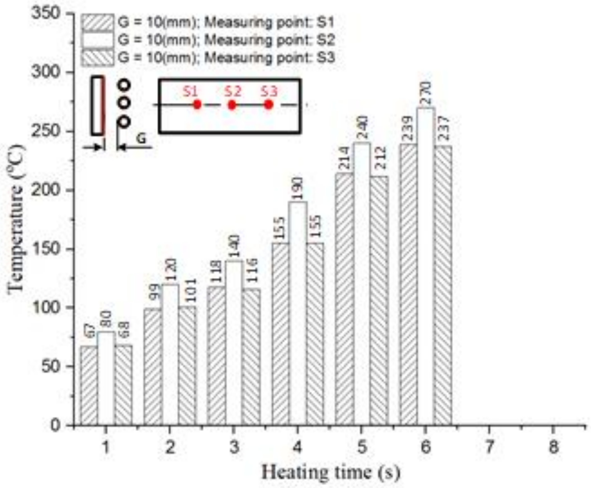

(b) The gap (G) of 10 mm

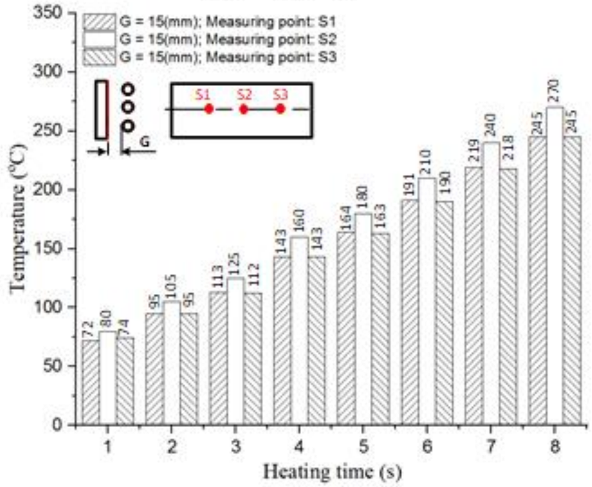

(c) The gap (G) of 15 mm

Fig 11. Temperatures at the measuring points (S1, S2, and S3) under different heating times and gaps (G)

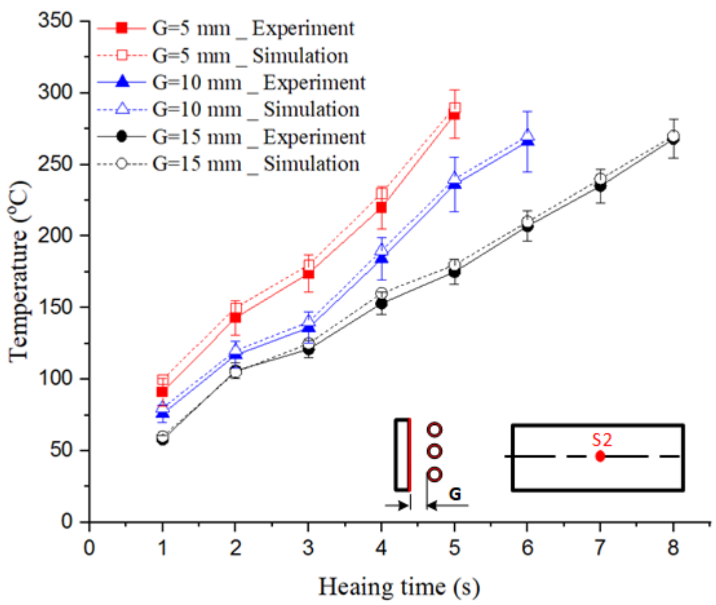

Fig 12: Temperature comparison between the experimental and simulated results at the center point (S2)

The temperature at S2 increased more rapidly and stabilized sooner than did those at S1 and S3. The experimental results showed that the temperature at S2 reached 295 °C after 5 s, and this value was approximately 15 °C higher than those measured at S1 and S3 (Fig 11). The simulation results exhibited a similar trend with a temperature deviation of less than 3%, compared with the experimental data (Fig 12). This difference is primarily because S2 is located in the central region of the mold, which features a thin-walled structure and is heavily influenced by heat transfer from the surrounding areas. In addition, the edge effect was stronger at S2, where stronger eddy currents accelerated the heating process. Achieving a high temperature at S2 is crucial to ensuring that the molten polymer can fully penetrate the thin-walled section. If the

temperature at S2 is insufficient, premature solidification may occur, leading to defects, such as short shots, weak weld lines, or incomplete filling in the final product.

(*) Comparison between simulated and experimental results

An analysis of both the simulated and experimental results showed that the temperature increase trends at the measurement points (S1, S2, and S3) were consistent. Specifically, the temperature at S2 increased the fastest and reached a stable value faster than did those at S1 and S3 because S2 was located centrally and was more influenced by edge effects. In contrast, the temperatures at S1 and S3 increased more slowly and remained relatively balanced, as these points were farther from the middle zone, leading to a weaker impact of the eddy currents on the heat distribution (Fig 11). The simulation results indicate that the temperature at S2 reached approximately 295 °C after 5 s, whereas at S1 and S3, the temperature ranged between 270 °C and 280 °C over a similar time frame. The experimental results showed similar trends, with S2 reaching between 293 °C and 297 °C while S1 and S3 ranged between 268 °C and 278 °C. Thus, these results demonstrate the high exactness of the simulation model (Fig 12).

The deviation between the simulated and experimental results was calculated to be less than 5% at almost every measurement point. Specifically, at S2, the deviation between the simulation and experiment ranged from 0.7% to 2%, indicating that the simulation model accurately captured the temperature increase in the central region. At S1 and S3, the deviation was slightly higher, ranging from 2% to 5%, mainly owing to experimental factors, such as convective heat losses, that were not fully considered in the simulation. Fig 11 illustrates the difference between the simulated and experimental temperatures over time at each measurement

point, and Fig 12 highlights the overall similarity in the temperature distribution on the mold surface.

There were three major reasons for the deviation between the simulation and experimental results. First, the heat losses in the experimental setup, including convection and radiation at the mold surface, were not completely accounted for in the simulation. This was particularly evident at S1 and S3, where the temperature rise was much slower. Second, measurement sensitivity and instrument accuracy contributed to the deviations. Devices such as thermocouples and infrared cameras introduced minor errors owing to measurement limitations, particularly at S2, where rapid temperature changes occurred. Finally, the simplifications in the simulation model directly affected the results. The simulation assumed constant material properties, such as thermal conductivity and density, whereas in reality, these properties vary with temperature and thereby impact the actual heat distribution on the mold surface.

Despite these deviations, the simulation method remained highly reliable for predicting the temperature distribution during the induction heating of the mold insert, providing a strong foundation for optimizing the manufacturing process. Furthermore, the results confirmed a high level of agreement between the simulation and experiment, with temperature deviations remaining below 5% at all measurement points, particularly at S2, which is the most important central region. The primary sources of deviation primarily stem from heat loss in the experimental setup and measurement inaccuracies. However, these factors do not significantly impact the accuracy or applicability of the simulation model in optimizing mold heating for the injection molding process.

Fig 13 illustrates the temperature change over time along Line L of the mold, which was measured experimentally at different heating distances (G = 5, 10, and 15 mm). Line L is a straight line that passes through the measurement points on the mold surface, including the middle region (S2) and the farthest regions (S1, S3). Hence, Fig 13 provides valuable information on the heating rate and the maximum temperature achieved at each heating gap G.

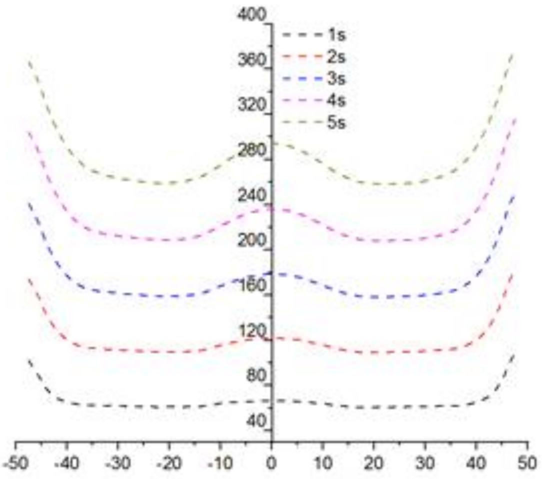

(a) Gap G = 5 mm

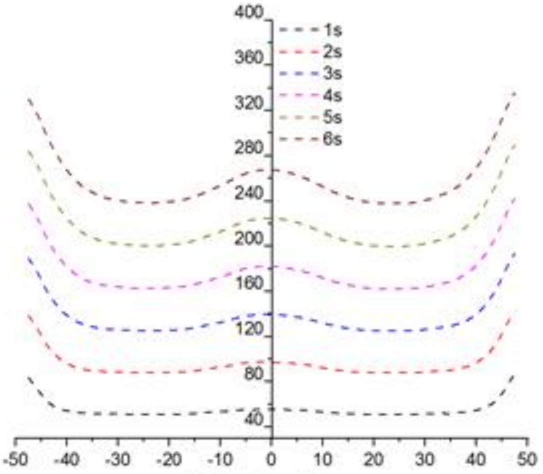

(b) Gap G = 10 mm

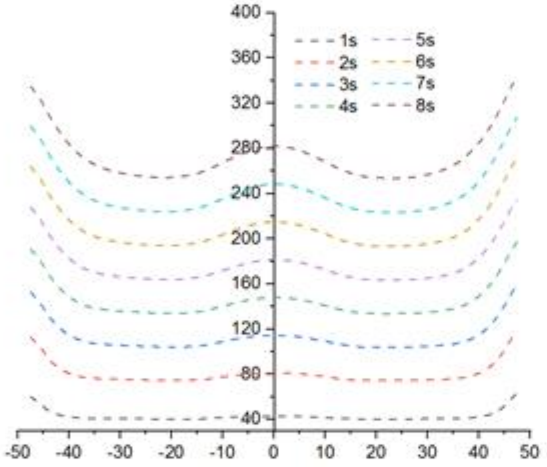

(c) Gap G = 15 mm

Fig 13: Time comparison of temperature on Line L with different heating distances (G) by experiment

Fig 13 illustrates the differences in the heating rate and temperature distribution along Line L when the heating distance G was varied. For G = 5 mm, the heating rate is highest because the temperature along Line L rapidly reached 295 °C within 5 s. This is because of the smaller space between the induction coil and the mold surface, which enhances the eddy current effect and improves the heat transfer efficiency. Remarkably, the temperature distribution along Line L was highly uniform, with less variation between the center and mold edges.

As the distance goes to G = 10 mm, the heating rate decreased slightly, with the temperature along Line L reaching 295 °C after approximately 6 s. The heat transfer efficiency decreased because of the larger gap, which weakened the magnetic field intensity. However, the temperature distribution remains relatively uniform, with a temperature difference of almost 10 °C between the middle zone and the mold edges. For G = 15 mm, the heating rate dropped significantly, with the temperature along Line L reaching 295 °C only at 8 s. At this larger distance, the lower heat transfer efficiency results in a strong temperature variation between the center and the mold edges, varying from 15 °C to 20 °C. These results indicate less heating uniformity, which could impact the consistency and quality of the molding process.

The simulation results clearly illustrate that a smaller heating distance G, such as 5 mm, provides the fastest heating rate and most uniform temperature distribution along Line L. When G was increased to 10 mm, the heating rate dropped slightly, but the target temperature remained within a reasonable timeframe, thus ensuring a balance between the heating efficiency and

operational security. At G = 15 mm, the heating time increased drastically; however, this setting is suitable for applications requiring higher safety levels or molds with longer production cycles.

The results in Fig 13 provide basic knowledge for optimizing the heating distance G in practical industrial applications. For G = 5 mm, the heating efficiency is superior, but careful control is required to prevent overheating or unintended contact between the induction coil and mold surface. A G of 10 mm offers a perfect balance between heating speed and operational safety, making it a better choice for most applications. Although G = 15 mm requires a longer heating time, it is suitable for molds requiring the safest levels or production cycles that allow for extended heating durations. Fig 13 shows that the heating speed and efficiency depend strongly on the distance G between the induction coil and the mold surface. A small distance guarantees faster heating and a more uniform temperature distribution, whereas a larger distance extends the heating time but enhances operational safety. The findings shown in Fig 13 provide a science-based approach for selecting the optimal G distance to ensure that it matches the technical requirements and specific characteristics of each production process.

3.3. Improvement of the tensile strength of weld lines

In injection molding, weld lines typically form when two molten polymer flows meet and solidify before merging. This phenomenon weakened the mechanical properties at the weld line locations and reduced the tensile strength of the final product. To address this issue, we applied external induction heating to increase the surface temperature of the mold cavity prior to polymer filling. Localized heating helps slow the cooling rate of the polymer when it contacts the mold and thus allows the molten state to be maintained for a longer duration. This extended melting

time enables better molecular bonding at the weld line, which leads to an improved microstructure and the enhanced mechanical strength of the final product [36].

Fig 14 illustrates the injection mold used for tensile testing, highlighting its key components: sprue, runner, gate, cavity, and thin-wall area. These elements play a crucial role in the injection molding process, directly influencing how the molten polymer fills the cavity, the formation of weld lines, and the overall quality of the final molded product. The cavity was the primary area of the mold, where the molten polymer was injected to form the test specimen. The cavity shape was designed according to tensile strength testing standards, featuring a narrowed central section to concentrate the stress during testing. This structural design accurately simulates the stress distribution when the product undergoes tensile loading and thus provides a reliable basis for evaluating the effects of induction heating on weld line quality.

The sprue serves as the main entry point for the molten polymer, feeding it from the injection nozzle into the mold. As the polymer flows into the mold, it passes through the runner, which is the channel system responsible for evenly distributing the molten polymer before it reaches the cavity. The runner was optimized to ensure a uniform flow distribution and thereby prevent pressure imbalances or uneven filling that could lead to defects. The gate was the final entry point before the polymer entered the cavity. In this mold design, two opposing gates were positioned to allow the two molten polymers to flow in opposite directions. This flow convergence in the central region results in the formation of a weld line, which is a critical area prone to weak bonding between the polymer streams or potential air entrapment, which can negatively impact the mechanical strength of the final product. Optimizing the heating process at this location can significantly improve the quality of the weld line.

The thin-section wall represents the thin-walled region of the mold, simulating the special injection molding conditions for thin-walled components. Filling this thin-walled region presents a significant challenge because the high cooling rate of the mold can cause premature solidification before complete filling. Without the assistance of a mold heating system, the mold-filling capability is significantly affected, leading to defects such as short shots or surface inconsistencies. In summary, the experimental mold shown in Fig 14 was specifically designed to simulate real-world injection molding conditions and assess the impact of induction heating on weld line strength. The use of two opposing gates ensures the formation of a weld line at the center of the specimen, which is a critical location for the evaluation of mechanical properties. By analyzing the data from this experiment, this study quantifies the improvements in tensile strength when applying induction heating, thus offering optimized solutions for manufacturing high-quality injection-molded products with enhanced mechanical durability.

.

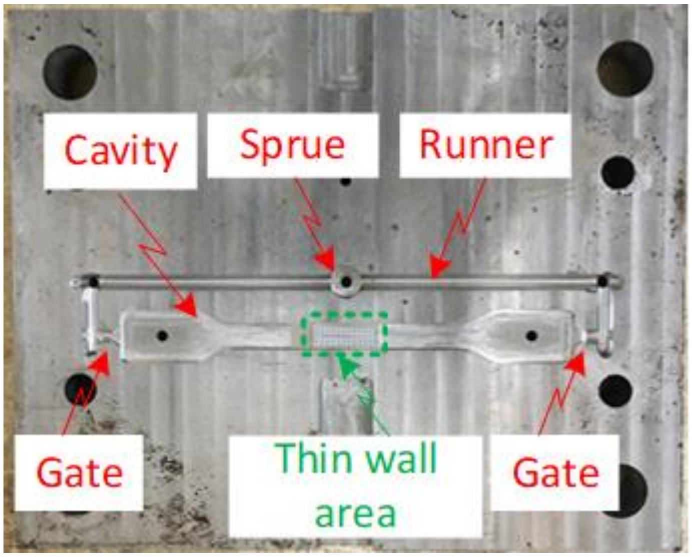

Fig 14. Tensile sample experiment mold

Increasing the mold temperature is a key factor in improving the quality of plastic products. A higher mold temperature decreases the temperature difference between the molten polymer and the mold surface and thereby minimizes the formation of the frozen layer. This effect facilitates a smoother polymer flow, allowing it to fill intricate and thin-walled sections more effectively, ultimately improving the mold-filling capability. The temperature distributions are illustrated in Figs 15, 16, and 17 and show that for heating distances of G = 5, 10, and 15 mm, the mold temperature increased as the heating time increased, thus contributing to a reduction in the frozen layer on the mold surface.

Weld lines typically form at the points where two molten polymer flows converge, and their quality is highly dependent on the mold temperature. When the mold temperature was low, the frozen layer formed more quickly, thus preventing the polymer from fully merging and resulting in a weak weld line. Conversely, higher mold temperatures, as observed in Fig 15 (G = 5 mm, with an extended heating time), allowing the polymer to remain in a molten state upon contact improves weld line bonding. Similarly, Figs 16 and 17 illustrate that although G = 10 mm or 15 mm requires a longer heating time, it is still possible to achieve sufficient temperature levels to enhance weld line quality while reducing weak bonding issues.

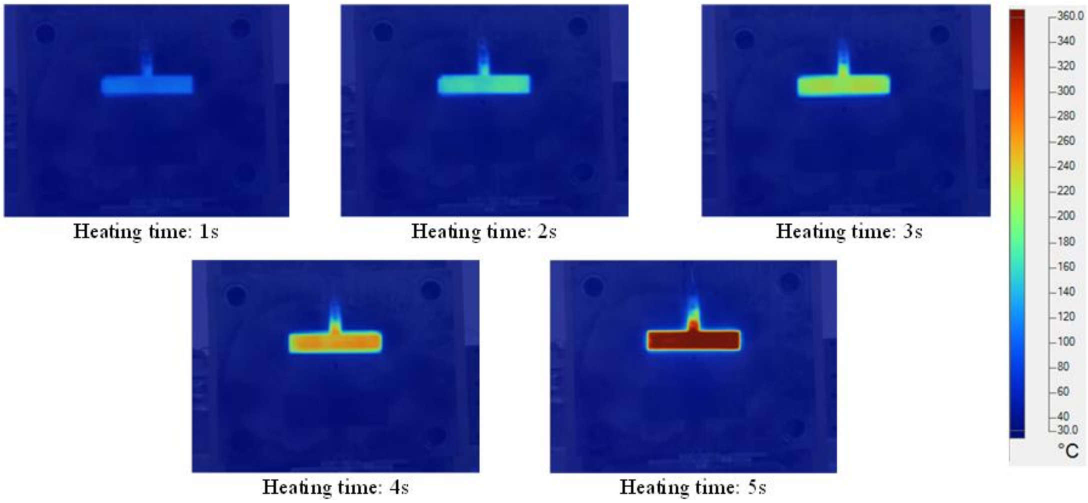

Fig 15. Temperature distribution of a cavity plate with different heating times under a gap (G) of 5 mm

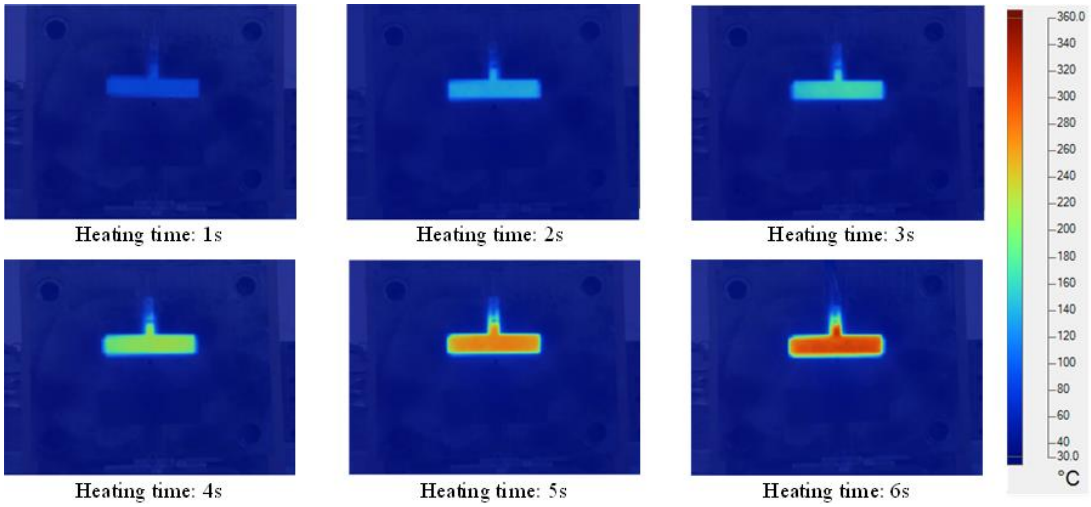

Fig 16. Temperature distribution of a cavity plate with different heating times under a gap (G) of 10 mm

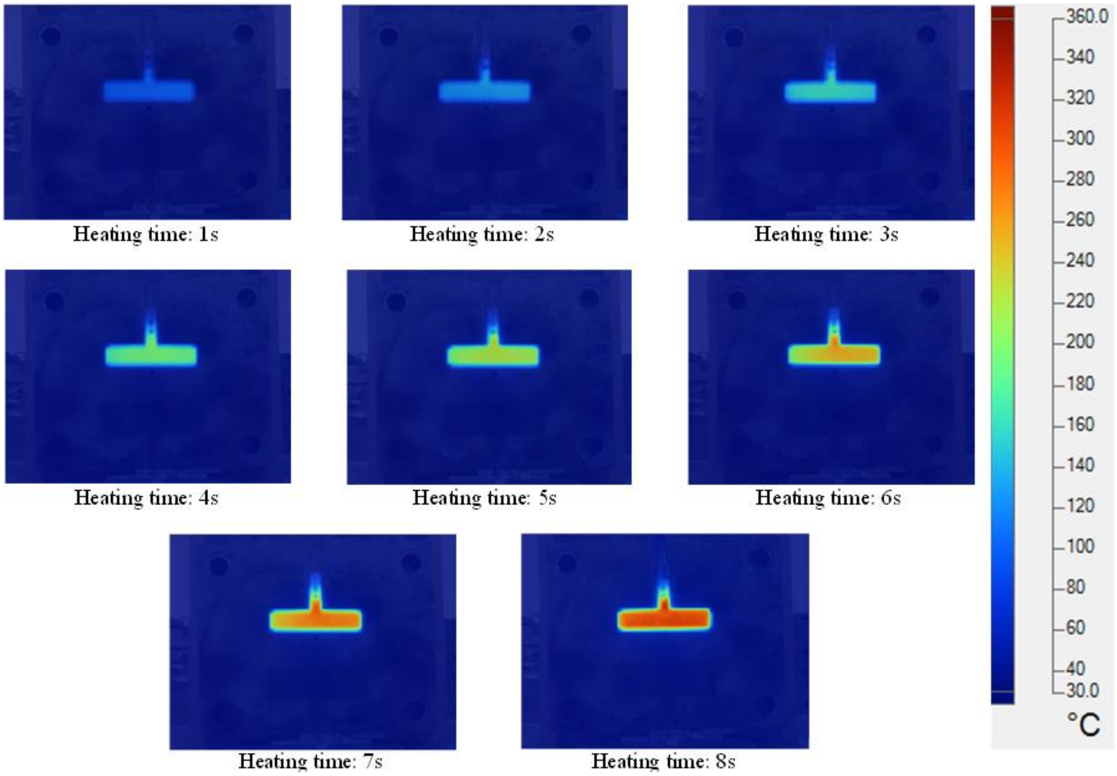

Fig 17. Temperature distribution of a cavity plate with different heating times under a gap (G) of 15 mm

At a heating distance of G = 5 mm, the heating process occurred rapidly, and the temperature was more evenly distributed across the mold surface, as illustrated in Fig 15. After 5 s of heating, the mold surface temperature reaches an optimal level of nearly 300 °C, which maintains the molten state of the polymer throughout the cavity-filling process. This uniform temperature distribution is particularly critical for minimizing weld line defects and improving the bonding quality at the polymer flow convergence points. As a result, the final product achieves a higher tensile strength and a reduced risk of defects such as air voids or the incomplete filling of thin-walled features.

At G = 10 mm, the heating rate was lower than that at G = 5 mm; however, it still ensured that the required mold surface temperature was reached, as shown in Fig 16. After 6 s of heating, the mold temperature ranges between 280 °C and 300 °C, which is sufficient to maintain the molten state of the polymer, particularly in the central region. Although the temperature distribution is slightly less uniform than that at G = 5 mm, it still contributes to an improved weld line quality, especially for high-precision molded products. The slightly extended heating time remained within an acceptable range for standard production cycles, thus balancing the heating efficiency and operational safety.

When the heating distance was increased to G = 15 mm, the time required to reach the optimal temperature increased significantly, as shown in Fig 17. After 8 s of heating, the mold temperature approaches 300 °C, which is sufficient to sustain the molten polymer state. However, the heating efficiency decreases owing to the greater distance, which weakens the magnetic field intensity. The temperature distribution becomes more uneven, with higher temperatures concentrated at the center of the mold. In contrast, the outer edges remain cooler. This setup is more suitable for applications with longer cycle times or those that prioritize operational safety over rapid heating.

In summary, the heating results shown in Figs 13, 15, and 16 confirm that a smaller heating distance (G = 5 mm) provides the fastest and most uniform heating, making it ideal for thin-walled components and high-quality molding requirements. However, longer distances (G = 10 and 15 mm) can still achieve the necessary mold temperature if the heating duration is appropriately adjusted, making them more suitable for longer production cycles or when operational safety is a higher priority. These findings provide an essential foundation for

optimizing the heating distance and duration in injection molding processes, thereby enabling manufacturers to enhance product quality while maintaining production efficiency and safety standards.

Fig 18 shows tensile test samples fabricated from different plastic materials, including PA6, PP, PC, and ABS. Each sample represents a commonly used industrial polymer, enabling the evaluation of tensile strength, mechanical durability, and the impact of various heating conditions on the mechanical properties of each material.

These data serve as a fundamental reference for analyzing the results in Fig 19, which shows the effects of the heating time and mold temperature on the tensile strengths of these materials and their long-term stability.

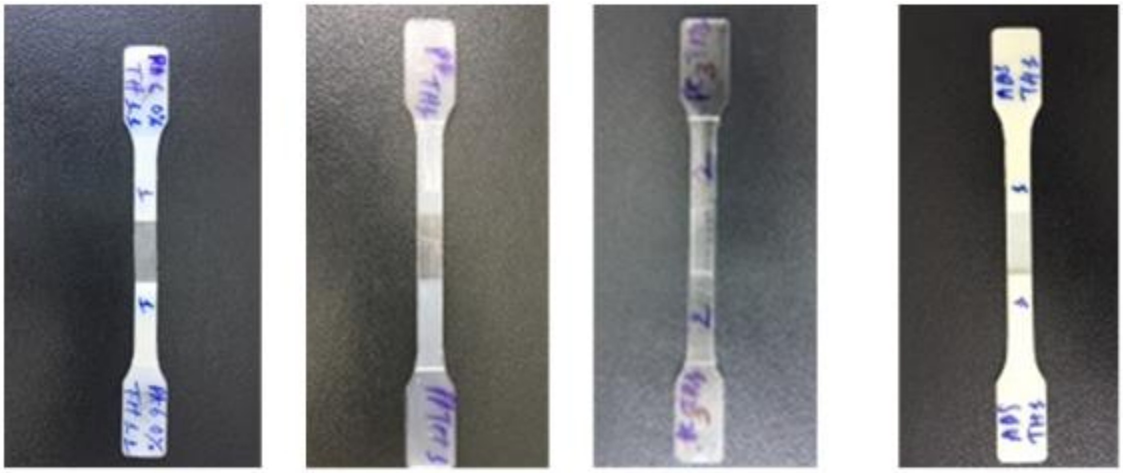

PA6 PP PC ABS

Fig 18. Tensile testing samples with different plastic materials

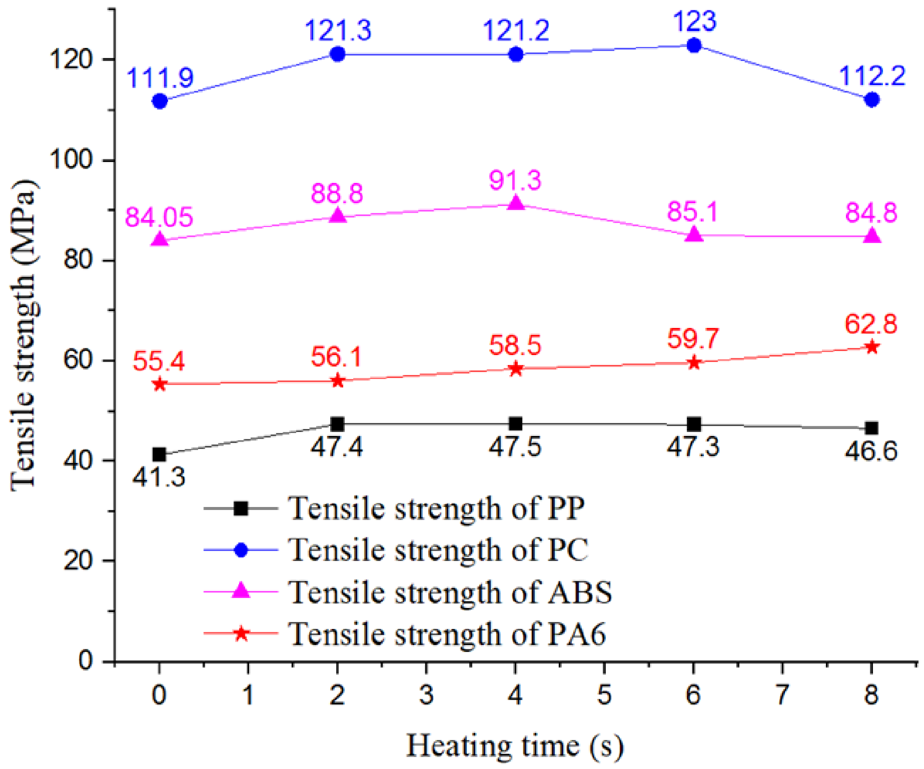

Fig 19. Effect of the heating time on the tensile strengths of different plastic materials under different heating times

The increase in the mold temperature not only improved the weld line quality but also significantly enhanced the tensile strength of the plastic products, as illustrated in Fig 19. The data in this figure demonstrate that as the heating time increased, the tensile strength of PC, ABS, PA6, and PP significantly improved.

For PC, the tensile strength increases from 111.9 MPa without heating (heating time = 0 s) to 123 MPa after 6 s of heating before slightly decreasing to 112.2 MPa when the heating time reaches 8 s. The ABS followed a similar trend, with the tensile strength increasing from 84.05 MPa (without heating) to a peak of 91.3 MPa after 4 s of heating but then declining as the heating time increased further.

For PA6, the tensile strength rises from 55.4 MPa without heating to 62.8 MPa at 8 s, demonstrating a clear positive effect of mold heating on this material. Last, PP, despite having a lower tensile strength than the other materials, still improves from 41.3 to 47.3 MPa when the heating time reaches 6 s. These improvements are primarily attributable to the higher mold temperatures, which reduce the frozen layer, improve the polymer flow into the mold, and enhance weld line bonding. However, when the mold temperature was too high, particularly when the heating time exceeded the optimal range, the tensile strength tended to decrease. For example, in PC, when heating for 8 s, the tensile strength drops from 123 to 112.2 MPa, whereas for ABS, the tensile strength decreases from 91.3 to 85.1 MPa when the heating time increases from 6 to 8 s. This phenomenon is attributable to the degradation caused by heat in the polymer or gas release within the material, which weakens the structure and reduces mechanical performance.

The study also revealed that without heating, the tensile strengths of all materials were significantly lower than those when mold heating was applied. Specifically, PC exhibited a tensile strength of 111.9 MPa, which was nearly 11 MPa lower than that measured when heated for 6 s. Similarly, ABS, PA6, and PP showed tensile strength reductions of approximately 7.25, 7.4, and 6 MPa, respectively, compared with their maximum values achieved with heating. This evidence strongly supports the conclusion that mold heating plays a crucial role in enhancing the mechanical properties of plastics.

Fig 19 shows that mold heating within the optimal time range (4–6 s) yields significant improvements in tensile strength without compromising product quality. However, the precise control of the heating time and temperature is essential to prevent excessive heating, which can

lead to material degradation or weakened weld line bonding. Furthermore, this study highlights that induction heating is an effective method by which to improve the quality of plastic products, not only by enhancing the tensile strength but also by reducing weld line defects. The collected data provide a scientific foundation for optimizing heating parameters, such as the heating distance (G) and duration, thus ensuring high production efficiency and a product of superior quality.

4. Conclusion

This study proposes a preheating method for the insert plate before placing it into the mold cavity, thus optimizing the cycle time without compromising product quality. Both the simulated and experimental results confirm that this approach effectively controls the mold temperature, which significantly enhances the tensile strength of plastic products and improves weld line quality by reducing the frozen layer during the mold filling process.

Experimental results across multiple plastic materials demonstrate that the tensile strength of PC increased from 111.9 MPa (without heating) to 123 MPa (with 6 s of heating), whereas ABS reached its peak tensile strength of 91.3 MPa after 4 s of heating. The PA6 and PP samples also exhibited substantial improvements in tensile strength when an appropriate heating duration was applied. However, when the heating time exceeded the optimal range (beyond 6–8 s), the tensile strength decreased owing to thermal degradation and gas release within the material.

Compared with traditional heating methods, such as resistance heating, steam heating, and hot gas heating (GMTC), the insert plate induction heating method significantly reduces the

waiting time, maintains a stable mold temperature, and does not extend the production cycle. Induction heating has proven to be an efficient and energy-saving solution with a fast heating rate, making it particularly suitable for thin-wall injection and microinjection molding, where precise temperature control is critical. Overall, this method not only enhances product quality but also optimizes the production efficiency and reduces the cycle time while ensuring superior mechanical properties in plastic products. Future studies should focus on further optimizing the heating system design and expanding its application to a wider range of plastic materials to ensure its adaptability to modern industrial requirements.

References

1. Kariminejad M, Tormey D, Ryan C, O'Hara C, Weinert A, McAfee M. Single and multi-objective real-time optimisation of an industrial injection moulding process via a Bayesian adaptive design of experiment approach. Sci Rep. 2024;14:29799. doi:10.1038/s41598-024-80405-2.
2. Sachs E, Wylonis E, Allen S, Cima M, Guo H. Production of injection molding tooling with conformal cooling channels using the three dimensional printing process. Polym Eng Sci. 2000;40:1232–47. doi:10.1002/pen.11251.
3. Xu X, Sachs E, Allen S. The design of conformal cooling channels in injection molding tooling. Polym Eng Sci. 2001;41:1265–79. doi:10.1002/pen.10827.
4. Dimla DE, Camilotto M, Miani F. Design and optimisation of conformal cooling channels in injection moulding tools. J Mater Process Technol. 2005;164–165:1294–300. doi:10.1016/j.jmatprotec.2005.02.162.
5. Kajikawa S, Iizuka T. Effect of molding temperature on fluidity and injection moldability of oven-dry steam-treated bamboo powder. J Mater Process Technol. 2015;225:433–8.
6. Gülçür M, Wilson P, Donnelly M, Couling K, Goodship V, Charmet J, et al. X-ray computed tomography for predictive quality assessment, 3D visualisation of micro-injection mouldings and soft-tool deformation. Mater Des. 2023;227:111741. doi:10.1016/j.matdes.2023.111741.
7. Li D, Zhang Y, Liu Y, Regi F, Brix Doest ME, Tosello G. Injection moulding of mechanical micro-manufactured structures for optically encoding plastic surfaces. Opt Mater. 2022;123:111822. doi:10.1016/j.optmat.2021.111822.

8. Liu J, Zhao B, Kontziampasis D, Jiang B, Wu W. A novel method for determining the pressure dependent characteristics of polymer melt during micro injection molding. Polym Test. 2024;137:108520. doi:10.1016/j.polymertesting.2024.108520.
9. Wang J, Dong H, Xu H, Fan F, Wu H, Qin Y, et al. Effect of jet milled ultrafine powder on porous tungsten emitter components by micro injection molding. Int J Refract Met Hard Mater. 2024;121:106659. doi:10.1016/j.ijrmhm.2024.106659.
10. Peixoto C, Valentim PT, Sousa PC, Dias D, Araújo C, Pereira D, et al. Injection molding of high-precision optical lenses: A review. Precis Eng. 2022;76:29–51. doi:10.1016/j.precisioneng.2022.02.002.
11. Ma Y, Li J, Jiao X, Jin B, Ye L, Yang W, et al. An injection molding method for optical lenses with high-quality consistency by regulating the closing behavior of non-return valve. Int J Adv Manuf Technol. 2024;135:5489–97. doi:10.1007/s00170-024-14786-z.
12. Dortu F, Bernier D, Cestier I, Vandormael D, Emmerechts C, Fissi LE, et al. Composite polymeric-inorganic waveguide fabricated by injection molding for biosensing applications. 16th International Conference on Transparent Optical Networks (ICTON); 2014 Jul; 2014. p. 1–4. doi:10.1109/ICTON.2014.6876325.
13. Wu Y, Peng W, Liu Y. A novel fabrication method for micro optical waveguide mold based on fly-cutting technology. Optik. 2013;124:867–9. doi:10.1016/j.ijleo.2012.02.020.
14. Mitrogiannopoulou AM, Tselepi V, Ellinas K. Polymeric and paper-based lab-on-a-chip devices in food safety: A review. Micromachines. 2023;14:986. doi:10.3390/mi14050986.

15. Saeedabadi K, Tosello G, Calaon M. Optimization of injection molded polymer lab-on-a-chip for acoustic blood plasma separation using virtual design of experiment. Procedia CIRP. 2022;107:40–5. doi:10.1016/j.procir.2022.04.007.
16. Macedo C, Freitas C, Brito AM, Santos G, Faria L, Laranjeira J, et al. Influence of dynamic temperature control on the injection molding process of plastic components. Procedia Manuf. 2019;38:1338–46. doi:10.1016/j.promfg.2020.01.155.
17. Seo J, Khajepour A, Huissoon JP. Thermal dynamic modeling and control of injection moulding process. Lect Notes Comput Sci. 2011:102–11. doi:10.1007/978-3-642-21538-4_11.
18. Kirchheim A, Katrodiya Y, Zumofen L, Ehrig F, Wick C. Dynamic conformal cooling improves injection molding. Int J Adv Manuf Technol. 2021;114:107–16. doi:10.1007/s00170-021-06794-0.
19. Yang H, Yilmaz G, Han G, Eriten M, Zhang Z, Yu S, et al. A quick response and tribologically durable graphene heater for rapid heat cycle molding and its applications in injection molding. Appl Therm Eng. 2020;167:114791. doi:10.1016/j.applthermaleng.2019.114791.
20. Yao D, Kim B. Development of rapid heating and cooling systems for injection molding applications. Polym Eng Sci. 2002;42:2471–81. doi:10.1002/pen.11133.
21. Liou AC, Chen RH, Huang CK, Su CH, Tsai PY. Development of a heat-generable mold insert and its application to the injection molding of microstructures. Microelectron Eng. 2014;117:41–47. doi: 10.1016/j.mee.2013.12.021.

22. Wang G, Zhao G, Li H, Guan Y. Research on a new Variotherm injection molding technology and its application on the molding of a large LCD panel. Polym Plast Technol Eng. 2009;48:671–681. doi: 10.1080/03602550902824549.

23. Wang G, Hui Y, Zhang L, Zhao G. Research on temperature and pressure responses in the rapid mold heating and cooling method based on annular cooling channels and electric heating. Int J Heat Mass Transf. 2018;116:1192–1203. doi: 10.1016/j.ijheatmasstransfer.2017.09.126.

24. Jeng MC, Chen SC, Minh PS, Chang JA, Chung C. Chung C. Rapid mold temperature control in injection molding by using steam heating. Int Commun Heat Mass Transf. 2010;37:1295–1304. doi: 10.1016/j.icheatmasstransfer.2010.07.012.

25. Guo Z, Xie J, Yang J, Fu J, Zhao P. Rapid mold temperature rising method for PEEK microcellular injection molding based on induction heating. J Mater Res Technol. 2023;26:3285–3300. doi: 10.1016/j.jmrt.2023.08.081.

26. Liparoti S, Sofia D, Pantani R. Rapid heating of mold: Effect of uneven filling temperature on part morphology and molecular orientation. Processes. 2023;11:273. doi: 10.3390/pr11010273.

27. Huang MS, Tai NS. Experimental rapid surface heating by induction for micro-injection molding of light-guided plates. J Appl Polym Sci. 2009;113:1345–1354. doi: 10.1002/app.30053.

28. Chen SC, Lin YW, Chien RD, Li HM. Variable mold temperature to improve surface quality of microcellular injection molded parts using induction heating technology. Adv Polym Technol. 2008;27:224–232. doi: 10.1002/adv.20133.

29. Yu MC, Young WB, Hsu PM. Micro-injection molding with the infrared assisted mold heating system. Mater Sci Eng A. 2007;460–461:288–295. doi: 10.1016/j.msea.2007.02.036.

30. Chang PC, Hwang SJ. Simulation of infrared rapid surface heating for injection molding. Int J Heat Mass Transf. 2006;49:3846–3854. doi: 10.1016/j.ijheatmasstransfer.2006.04.014.

31. Gao S, Qiu Z, Ma Z, Yang Y. Development of high efficiency infrared-heating-assisted micro-injection molding for fabricating micro-needle array. Int J Adv Manuf Technol. 2017;92:831–838. doi: 10.1007/s00170-017-0169-5.

32. Simoes R, Macedo C, Laranjeira J, Brito AM, Simões CL, Faria L. Development of a rapid heat cycle injection molding system using infrared radiation and convection heating and influence on morphology and mechanical properties. Int J Adv Manuf Technol. 2024;130:283–295. doi: 10.1007/s00170-023-12683-5.

33. Do TT, Uyen TMT, Minh PS. The feasibility of an internal gas-assisted heating method for improving the melt filling ability of polyamide 6 thermoplastic composites in a thin wall injection molding process. Polymers. 2021;13:1004. doi: 10.3390/polym13071004.

34. Minh PS, Do TT, Uyen TMT. The feasibility of external gas-assisted mold-temperature control for thin-wall injection molding. Adv Mech Eng, Internet. 2018;10:168781401880610. doi: 10.1177%2F1687814018806102.

35. Uyen TMT, Do TT, Minh PS. Internal gas-assisted mold temperature control for improving the filling ability of polyamide 6 + 30% glass fiber in the micro-injection molding process. Polymers. 2022;14:2218. doi: 10.3390/polym14112218.

36. Minh PS, Uyen TMT, Do TT, Nguyen VT, Nguyen VTT. Enhancing the Fatigue Strength of the Weld Line in Advanced Polymer Injection Molding: Gas-Assisted Mold Temperature Control for Thermoplastic Polyurethane (TPU) Composites. Polymers. 2023;15:2440–2440. doi: 10.3390/polym15112440.

37. Rudnev V, Loveless D, Cook R, Black M. Handbook of induction heating. New York: Marcel Dekker; 2003.

38. Tavakoli MH, Karbaschi H, Samavat F. Influence of workpiece height on the induction heating process. Math Comput Modell. 2011;54:50–58. doi: 10.1016/j.mcm.2011.01.033.
