# Supplementary material for: Applying a new mold temperature control strategy to improve the tensile strength of thin wall products in injection molding processes
Source: PLoS One. 2025 Dec 5;20(12):e0337889. doi: 10.1371/journal.pone.0337889 (PMC12680320; doi:10.1371/journal.pone.0337889)
Supplement: Attachment — Submitted filename: PONE-D-25-07599_Cover letter to Reviewer 4.pdf [file pone.0337889.s004.pdf]

Subject: *Point-by-Point Response to Reviewer 4 for Revised Manuscript PONE-D-25-07599: Applying a new mold temperature control strategy to improve the tensile strength of thin wall products in injection molding processes*

Dear Reviewer 4,

We are sincerely grateful for your positive assessment of our manuscript as an "interesting paper" and for the thoughtful comments that have helped us enhance its quality. Your recognition of the innovative approach to improving weld line strength through induction heating is greatly appreciated, and we value the time and expertise you dedicated to providing constructive feedback. Your suggestions have guided us in addressing key areas, and we are committed to making the necessary improvements. Below, we offer a point-by-point response to each of your comments, detailing the revisions made. All changes are highlighted in the revised manuscript via inline comments for your convenience, with line references to the updated version. We hope these updates fully address your concerns and elevate the manuscript's impact. Your insights have been invaluable, and we welcome any additional guidance you may wish to share.

Reviewer 4 Comment 1: Although this idea of induction heating is great, however, must pay attention to the mold insert material if it can sustain the thermal fatigue.

➔ **Reply:** Thank you for the comment. We acknowledge the importance of considering the mold insert material's ability to withstand thermal fatigue under induction heating. In this study, materials with sufficient thermal resistance were selected, and further durability assessments are recommended for long-term applications (lines 450–453). This addition reflects your concern and ensures a balanced discussion on material suitability, and we are grateful for your foresight in highlighting this critical aspect.

Reviewer 4 Comment 2: How many tensile bars were tested? If more than one, tensile strength should have STD on Figure 19.

➔ **Reply:** We thank you for raising this important point, which ensures the reliability of our mechanical data. In this study, 10 tensile bars were tested, and the average tensile strength was used for comparisons. Figure 19 has been updated to include the standard deviation (STD) to reflect the variability among the tested samples (lines 710–712). This revision enhances the statistical rigor of our results, and your suggestion has significantly improved their presentation.

Reviewer 4 Comment 3: Unit of viscosity should be Pa*s, density should be kg/m^3, ^{3} is superscript.

➔ **Reply:** Thank you for catching this oversight, which is essential for maintaining scientific accuracy. We have modified the units accordingly: viscosity is now listed as Pa·s and density as kg/m^3 with the appropriate superscript (lines 160–165). This correction applies to Table 2 and related text, ensuring consistency, and we appreciate your keen eye for detail.

Reviewer 4 Comment 4: Can you supply color figures on Figures 8, 9, and 10. What simulation software you used? Can not distinguish the temperature level on your figures. Red color means hot and blue color means cool on color figures.

➔ **Reply:** We are grateful for your feedback on improving figure clarity, which is vital for effective data interpretation. Figures 8, 9, and 10 are now provided in color, with red indicating higher temperatures and blue indicating lower temperatures, as per your suggestion (lines 390–395). We used COMSOL Multiphysics for simulating the induction heating process and Moldex3D for molding process parameters, which we have clarified in the text (lines 130–135). This update should enhance readability, and thank you for guiding us toward a more distinguishable presentation.

Reviewer 4 Comment 5: Resolution on Figures 11 and 13 are poor.

➔ **Reply:** Thank you for pointing out this issue, which affects the visual quality of our data. We have improved the resolution of Figures 11 and 13 to ensure clearer and more detailed visuals (lines 400–402). This enhancement improves the figures' usability, and we value your input in refining them.

Reviewer 4 Comment 6: Caption should be "The weld-line strength", do you use one gate or two gate? If two gates, must use weld-line strength on Figure 19.

➔ **Reply:** We appreciate your suggestion for improving caption accuracy and your focus on experimental design. We have modified the caption for Figure 19 to read "…tensile strengths of weld-line…" (line 715), reflecting the two-gate configuration used in our experiments, which naturally forms weld lines. This adjustment aligns with the study's focus on weld line strength, and thank you for helping us clarify this detail.

Reviewer 4 Comment 7: Heating time on Figure 12.

➔ **Reply:** Thank you for identifying this typographical error, which ensures the accuracy of our figure labels. We have corrected the error on Figure 12, properly labeling the x-axis as "Heating Time" (line 434). This small fix enhances clarity, and we are grateful for your careful review.

We are deeply grateful for your contributions, which have strengthened the manuscript and addressed its key areas for improvement. Should you require any further clarifications or adjustments, please do not hesitate to let us know—we are committed to incorporating your wisdom fully.

Sincerely,

Duc Thuan Huynh (corresponding author) Ho Chi Minh City University of Technology (HCMUT) hdthuan.sdh241@hcmut.edu.vn +84989961299

On behalf of the co-authors: Thanh Trung Do, Tran Anh Son, Pham Son Minh
