# Supplementary material for: Applying a new mold temperature control strategy to improve the tensile strength of thin wall products in injection molding processes
Source: PLoS One. 2025 Dec 5;20(12):e0337889. doi: 10.1371/journal.pone.0337889 (PMC12680320; doi:10.1371/journal.pone.0337889)
Supplement: Attachment — Submitted filename: PONE-D-25-07599_Cover letter to Reviewer 4.docx [file pone.0337889.s005.docx]

Subject: *Point-by-Point Response to Reviewer 1 for Revised Manuscript PONE-D-25-07599: Applying a new mold temperature control strategy to improve the tensile strength of thin wall products in injection molding processes*

Dear Reviewer 4,

Thank you very much for the helpful feedback and for the opportunity to revise our manuscript. We truly appreciate the time and effort that you dedicated to evaluating our work.

We would like to confirm that we have carefully addressed all of your comments. In particular:

1. **All requested corrections to units and symbols have been made.**
   - Viscosity units have been corrected to **Pa·s**.
   - Density units have been revised to **kg/m³**, with the superscript properly formatted.
   - We also checked the entire manuscript to ensure consistency in scientific notation and units.
2. **All additional suggestions have been carefully considered and implemented to improve clarity and presentation.**
3. **To facilitate the review process, we have clearly highlighted all revisions in the “Revised Manuscript with Track Changes” file.**

We appreciate your suggestions about using colored text or a marked version to show modifications, and we have followed this recommendation in this revision to save time for reviewers.

A detailed point-by-point “Response to Reviewers” document has been prepared and uploaded, together with both the tracked-changes and clean versions of the revised manuscript.

We sincerely thank you for the constructive feedback, which helped us improve the quality and clarity of the manuscript. Please let us know if any further changes are required.

Kind regards,

Duc Thuan Huynh (corresponding author) Ho Chi Minh City University of Technology (HCMUT) [hdthuan.sdh241@hcmut.edu.vn](mailto:hdthuan.sdh241@hcmut.edu.vn) +84989961299

On behalf of the co-authors: Thanh Trung Do, Tran Anh Son, Pham Son Minh
